# REvolutionH-tl 2.0: A fast and robust tool for decoding evolutionary gene histories

**DOI:** 10.1371/journal.pcbi.1013017

**Published:** 2026-08-12

**Authors:** José Antonio Ramírez-Rafael, Annachiara Korchmaros, Katia Aviña-Padilla, Alitzel López-Sánchez, Gabriel Martinez-Medina, Alfredo J. Hernández-Álvarez, Marc Hellmuth, Peter F. Stadler, Maribel Hernández-Rosales

**Affiliations:** 1 Department of Genetic Engineering, Center for Research and Advanced Studies of the National Polytechnic Institute, Irapuato Unit, Irapuato, Guanajuato, Mexico; 2 Department of Computer Science, University of Leipzig, Leipzig, Saxony, Germany; 3 Max Planck Institute for Mathematics in the Sciences, Leipzig, Saxony, Germany; 4 Department of Cell Biology, Center for Research and Advanced Studies of the National Polytechnic Institute, Mexico City, Mexico; 5 Department of Computer Science, University of Sherbrooke, Sherbrooke, Quebec, Canada; 6 Centro de Ciencias Genómicas, UNAM, Cuernavaca, Mexico; 7 Department of Mathematics, Stockholm University, Stockholm, Sweden; 8 Department of Theoretical Chemistry, University of Vienna, Vienna, Austria; 9 Santa Fe Institute, Santa Fe, New Mexico, United States of America; 10 Facultad de Ciencias, Universidad Nacional de Colombia, Bogotá, Colombia; University of Toronto, CANADA

## Abstract

REvolutionH-tl is a fast, scalable, and integrated software platform for inferring orthology relationships, gene trees, species trees, and reconciled evolutionary scenarios directly from sequence data. Built upon the formal framework of best match graphs (BMGs), REvolutionH-tl predicts orthogroups and orthologous gene pairs with high accuracy, requiring neither precomputed trees nor multiple external tools. The software reconstructs event-labeled gene and species trees, seamlessly integrating reconciliation to produce fast, accurate, and biologically insightful evolutionary scenarios. Through extensive benchmarking on synthetic datasets with known ground truth, REvolutionH-tl outperforms or matches the accuracy of established tools such as OrthoFinder, Proteinortho, RAxML, GeneRax, and RANGER-DTL, while achieving significantly lower runtimes. A key innovation of REvolutionH-tl is its built-in support for detailed, publication-ready visualizations, which allow users to explore genome evolution dynamics, orthogroup composition, and reconciliation results with clarity and ease. These visual features position REvolutionH-tl as the first platform of its kind to combine analytical precision with intuitive interpretability. The software is open-source, cross-platform, and freely available at https://pypi.org/project/revolutionhtl/, providing a robust solution for large-scale evolutionary analyses in comparative genomics.

## Author summary

Comparative genomics relies on understanding how genes evolve across species. This involves identifying groups of related genes, reconstructing their evolutionary trees, and aligning them with the evolutionary history of species. These steps are typically performed using multiple tools, often requiring manual integration and technical expertise. We present REvolutionH-tl, an open-source software that automates the entire evolutionary reconstruction process—starting from protein sequences and producing gene trees, species trees, orthology assignments, and reconciled evolutionary scenarios. Unlike existing tools, REvolutionH-tl also includes built-in, high-quality visualizations that help users interpret complex evolutionary events such as gene duplications and losses. We evaluated REvolutionH-tl on simulated genomes with known evolutionary histories and found that it matches or exceeds the performance of widely used tools, while being significantly faster. Its visual output makes evolutionary analysis more accessible and interpretable, offering a valuable resource for researchers studying genome evolution.

## Introduction

Phylogenetics reconstructs evolutionary histories by tracing shared characteristics in descendant lineages back to a common ancestor. The best-supported hypotheses of evolutionary histories are usually represented with phylogenetic trees, where each node depicts the emergence of a new lineage, while each branch represents the preservation of a genetic lineage over time. In particular, if a tree represents the evolutionary relationship among a group of species, then the branching points represent a speciation event. On the other hand, if a tree represents the evolution of genes, then the internal nodes represent speciation, duplication, or other events the genes went through [1].

Reconstructing the evolutionary history between genomic entities helps to understand the evolution of morphological characteristics, reconstruct demographic changes in recently diverged species, and infer, in particular, the function of newly discovered genes. Functional gene annotation relies on identifying which homologous genes are orthologs, as they tend to preserve functions that were present in their ancestors [2]. In this context, a *gene* represents either a nucleotide or amino acid sequence, and a set of homologous genes share a common ancestor. Two genes are *orthologs* if they diverge from a single ancestral gene amid the speciation process of the last common ancestor of the species in which such genes reside.

Although genetic material may be transferred between species, giving rise to xenologous genes, in this contribution we assume the evolutionary histories are free of horizontal gene transfers (HGT), i.e., the nodes in the gene tree represent either duplication or speciation events. We refer the reader to [3] for further details on gene transfers. Moreover, our approach aims to reconstruct macro-evolutionary relationships among genes and species, focusing on speciation and gene duplication events represented in phylogenetic trees. Population-level processes such as incomplete lineage sorting (ILS), which is the preservation of gene polymorphisms during a speciation event, are not explicitly modeled. These kinds of evolutionary processes take less relevance in this study, as the time between speciation events increases relative to the effective population size of ancestral species, and thus becomes less influential over longer evolutionary timescales relevant to macroevolutionary inference [4].

With increasing attention regarding genome and transcriptome annotation, the development of computational methods to infer orthologous relations and reconstruct the gene and corresponding species trees have gained considerable interest [5,6]; despite the increasing availability of phylogenetic data, the process of accurately reconstructing evolutionary histories is not always straightforward. Methods for orthology detection are either tree-based or graph-based. Tree-based methods identify orthologs by reconciling an explicit tree model of the genes’ history to one of the species where they reside. Orthologs are then set to be genes that group with members from other species in the tree reconciliation, as in [5]; in [7] this approach is formalized in an algorithm that assigns gene duplication and speciation events in polynomial time. In principle, tree-based methods are the most appropriate for orthologous detection; however, such methods are precluded when the number of leaves is large, as trees are computationally expensive to produce [5].

Our main interest is in graph-based orthology methods, which model homologous genes as nodes in a graph and infer orthology relations as edges based on sequence similarity and the assumption of underlying gene and species trees. In scenarios without horizontal gene transfer, orthologous genes are expected to be *best matches*, meaning homologs from different species whose lineages diverged most recently. However, the converse is not guaranteed: gene loss can lead to situations in which two genes are best matches even though they are not orthologs; such genes diverged by a duplication event followed by speciation and differential gene loss in the descendant species [8].

Moreover, if evolutionary closeness is estimated by means of sequence similarity, orthologs are detected as *best hits*, i.e., the genes with the highest similarity score match. Pairwise sequence similarity scores are usually computed as bit-scores using all-vs-all BLAST search [9] or DIAMOND [10,11] as an accelerated alternative to BLAST. It is worthy to highlight the difference between a best match and a best hit: the former is a theoretical relation based on the assumption that two genes belong to different species in the same species tree, while the later is a data-driven approximation.

Bidirectional best hits is the most widely used graph-based approach to orthology inference. The method is based on the assumption that orthology is a reciprocal evolutionary relationship: two genes are inferred to be orthologs if each is the best scoring hit for the other in different species. This approach limits one ortholog per gene and species, which is false when a gene undergoes duplication after speciation. Methods that allow multiple orthologs (co-orthologs) are available; in particular, ProteinOrtho [12] selects the orthologs whose score is above a dynamical score that depends on the gene and species considered. Moreover, “bidirectional best hits” is theoretically incomplete: even when the inference of bidirectional best hits coincides exactly with the true bidirectional best matches for a collection of genes, these relations only form a superset of orthologs and thus require further processing [13,14]. Refer to S1 Appendix for examples. Graph-based approaches are computationally efficient and scale well with large datasets compared to tree-based approaches. However, as in tree-based approaches, orthologs paired with reconciled gene trees provide more information to the user than sets of orthologs. An alternative to the classical methods for assessing orthology in sequenced transcriptomes is comparing newly discovered sequences to a set of reference orthologs from dedicated databases [15].

Phylogeny reconstruction methods are either distance-based or character-based. Distance-based methods iteratively build a tree using a distance matrix between every pair of taxa. Neighbour joining [16] is the most popular of such methods, and recent implementations typically run approximately to the square of the number of taxa; however, the correctness of the output relies on an additive and unbiased distance matrix, which cannot be guaranteed. Maximum parsimony (MP), maximum likelihood (ML), and Bayesian inference (BI) are the main types of character-based methods [17]. BI and ML rely on an explicit evolutionary model and the likelihood function L(θ), which is the probability of observing the data given that the parameter θ includes a substitution model and branch length parameters. The ML tree estimates the topology with the highest likelihood. BI tree estimate is instead the maximum posterior probability tree among all topologies simulated via the Markov chain Monte Carlo algorithm, where the posterior probability is obtained by multiplying a prior distribution and the likelihood. To avoid parameter estimations of a given evolutionary model, recently, phylogenetic invariants have been used to estimate the tree topology [18,19]. All these methods are computationally expensive and require specific evolutionary model assumptions, which may negatively affect the tree estimate. On the other hand, the maximum parsimony (MP) approach, which is the one of interest in this paper, is not susceptible to the drawbacks of likelihood-based models; indeed, MP tree estimation involves the minimum number of events among the taxa. However, MP is more susceptible to the long-branch attraction artifact (LBA) in the case of heterogeneous rates [20].

The practical superiority of graph-based methods over phylogenetic approaches for the accurate prediction of orthology relations motivated a recent mathematical interest [21–25] in detecting orthology through best match graphs (BMGs), which group the most closely related genes between species in directed graphs called BMGs. In [26], a theoretical pipeline for inferring orthologous relationships and tree phylogenies was proposed but remained unimplemented until recently. Ramírez-Rafael et al. [27] introduced REvolutionH-tl (**R**econstruction of **Evolution**ary **H**istories **t**oo**l**), a novel bioinformatics tool that applies the BMG theoretical framework to identify orthologous genes and groups (orthogroups) and to reconstruct and reconcile event-labeled gene and species trees in the presence of duplications and losses.

REvolutionH-tl belongs to the family of graph-based orthology methods, initially constructing a BMG estimate from best-hit data (e.g., BLAST, Diamond). Gene and species trees are inferred by maximizing the number of informative triples—BMG building blocks—without relying on explicit evolutionary models, aligning the method with maximum parsimony principles. Reconciliation is performed by mapping duplications and losses onto species tree branches, while contradictions between gene and species trees prompt alternative hypotheses through gene tree editing, enabling the detection of pseudo-orthologs and pseudo-paralogs.

Here, we present a new version, REvolutionH-tl 2.0, an enhanced platform of the original tool that incorporates improved components and data structures, resulting in significantly faster performance. In addition, it introduces a modified neighbor-joining method to resolve duplication nodes in gene trees. This new version also includes a comprehensive set of visualizations designed to facilitate more user-friendly analysis and interpretation of results.

We evaluated REvolutionH-tl 2.0 against established tools such as ProteinOrtho [12,28,29], OrthoFinder [30,31], GeneRax [32], ASTRAL-Pro [33], RAxML [34], and RANGER-DTL [35]. Our benchmarking demonstrates that most of the time REvolutionH-tl outperforms other methods in orthology inference, gene and species tree reconstruction while significantly reducing computational time. Furthermore, we assessed the precision of our orthology predictions using the *Quest for Orthologs benchmark service* [36], confirming the strength of REvolutionH-tl for prediction of precise orthology relations with meaningful biological information. Its combination of accuracy, efficiency, and scalability makes REvolutionH-tl a powerful tool for evolutionary analyses, helping researchers uncover the complexities of gene and species evolution with precision and ease.

## Theoretical background

### Graph theory preliminaries

A *graph G* is an ordered pair *G* = (*V*, *E*), where *V* is the set of *nodes* and E⊆{uv∣u,v∈V,u≠v} is the set of *edges*, representing connections between nodes. If uv∈E implies vu∈E for all u,v∈V, we call *G undirected* and, otherwise, *directed*.

The *degree* of a node v∈V in an undirected graph is the number of edges incident to *v*. In a digraph, the *in-degree* and *out-degree* of *v* refer to the number of incoming edges *uv* and outgoing edges *vu*, denoted $\deg_{G}^{-}(v)$ and $\deg_{G}^{+}(v)$, respectively. A *subgraph H* of *G* is a graph such that V(H)⊆V(G) and E(H)⊆E(G). The *induced subgraph* of *G* on a node set $V^{\prime }\subseteq V(G)$, denoted $G[V^{\prime }]$, is the subgraph $H=(V^{\prime },E^{\prime })$, where $E^{\prime }=\{uv\in E(G)|u,v\in V^{\prime }\}$ contains all those edges from *E*(*G*) that connect pairs of nodes in $V^{\prime }$.

A *directed acyclic graph (DAG)* is a directed graph without directed cycles. A *rooted tree T* is a DAG that does not contain nodes *v* with $\deg_{G}^{-}(v)>1$ and that has a unique *root* $\rho_{T}$, i.e., a vertex $\rho_{T}$ with $\deg_{G}^{-}(v)=0$ and from which all nodes *v* are reachable via a directed path from $\rho_{T}$ to *v*. By definition, if |*V*| > 1, then for each $v\in V⧵\{\rho_{T}\}$ there is a unique incoming edge *uv* and we call *u* the *parent* of *v* and *v* a *child* of *u*. We collect in $child_{T}(u)$ all childen of *u* in *T*. The nodes of a rooted tree can be classified as *leaves*, *L*(*T*), which are terminal nodes (nodes with no children), and *internal nodes*, *V*^0^(*T*), which have at least one child. If there is a directed path from *u* to *v*, then *u* is an *ancestor* of *v* and *v* a *descendant* of *u*, denoted by $v{⪯}_{T}u$. We write u∥v if neither $v{⪯}_{T}u$ nor $u{⪯}_{T}v$ holds and say that *u* and *v* are *incomparable* in *T*. Furthermore, in [37,38] the ${⪯}_{T}$ relationship has been extended to consider edges within *T* as follows. Let *e* = *uv* be an edge and *x* be a node in *T*. Then, we put $x{≺}_{T}e$ if and only if $x{⪯}_{T}v$. Moreover, we put $e{≺}_{T}x$ if and only if $u{⪯}_{T}x$. For edges *e*=(*u*,*v*) and *f*=(*a*,*b*) in *T* we put $e{⪯}_{T}f$ if and only if $v{⪯}_{T}b$.

For any node v∈V(T), the *subtree rooted at v*, denoted *T*(*v*), is the subgraph of *T* induced by *v* and all its descendants. For any node v∈V(T) in a rooted tree *T*, the *cluster* of *v*, denoted *C*(*v*), is the set of leaves in the subtree rooted at *v*, i.e., *C*(*v*) = *L*(*T*(*v*)). The *last common ancestor* (LCA) of a set X⊆V(T), denoted $lca_{T}(X)$, is the node in *T* that is an ancestor of all nodes in *X* and none of the nodes $w{≺}_{T}lca_{T}(X)$ satisfy this property. If *X* ={*x*,*y*} we write $lca_{T}(x,y)$ instead of $lca_{T}(\{x,y\})$.

A *phylogenetic tree* is a rooted tree where each internal node has at least two children. Since gene duplications, losses or other events may predate the root of a phylogenetic tree (in particular, in the context of tree reconciliations [8]), we define a *planted tree* that is formed by adding a new node $0_{T}$ to a phylogenetic tree *T* and an edge $0_{T}\rho_{T}$. In a planted tree, the node $0_{T}$ has degree one, with its only neighbor being $\rho_{T}$, and this property remains unchanged during any subsequent modifications, such as resolving ’polytomies’ as explained next. A *polytomy* in a phylogenetic tree is a node with more than two children. Resolving a polytomy involves converting it into a binary tree, a tree where each internal node has exactly two children. This may introduce additional internal nodes to maintain the tree’s structure. Given a gene tree *T* and a subset of leaves $L^{\prime }\subseteq L(T)$, the *restriction* $T{|}_{L^{\prime }}$ of *T* to the set $L^{\prime }$ is obtained from the minimal subtree of *T* connecting all the leaves in $L^{\prime }$ by suppressing all the vertices with a single child.

### Triples, the Aho graph and BUILD

We now introduce the concept of triples, which form the fundamental basis for inferring gene and species trees directly from genomic sequence data. A *triple xy*|*z* is a phylogenetic tree *T* on three leaves *x*, *y*, and *z* such that $lca_{T}(x,y){≺}_{T}lca_{T}(\{x,y,z\})$. Similarly, a rooted tree *T displays* a triple *xy*|*z* if $lca_{T}(x,y){≺}_{T}lca_{T}(\{x,y,z\})$. Such triples are crucial for reconstructing phylogenetic trees; see, e.g., [39–41]. For any rooted tree *T*, let *R*(*T*) denote the collection of triples displayed by *T*. An arbitrary set *R* of triples is *compatible* if there exists a rooted tree *T* such that R⊆R(T). A polynomial-time procedure to verify the compatibility of a set *R* and, if compatible, to construct a tree that displays all triples in *R* is provided by the BUILD algorithm [39,42]. This algorithm constructs an auxiliary graph $G_{R,L}$, commonly referred to as the *Aho graph*, where the nodes correspond to all leaves in *L*, and edges *xy* exist for each triple xy|z∈R with x,y,z∈L. This algorithm begins with the set *L* of all leaves in *R* and recurses on the connected components $L_{1},...,L_{k}$ of the Aho graph, repeating the process for the graphs $G_{R,L_{1}},...,G_{R,L_{k}}$. If, at any step, one of the Aho graphs $G_{R,L^{\prime }}$ is connected and contains more than one node, the initial set *R* is declared as *not compatible*. In the following, we let Aho(*R*) be the function that takes as input a triples set *R*, and outputs either the tree *T* constructed by BUILD or the sentence *the set of triples R is not compatible*.

### Evolutionary scenarios

In what follows we consider particular types of phylogenetic trees, namely species trees and gene trees. A *species tree S* = (*V*, *E*) represents the evolutionary history of a set of species, where the leaves L(S)⊆V(S) correspond to extant species. A *gene tree T* = (*V*, *E*) represents the evolutionary history of a set of genes, where the leaves L(T)⊆V(T) correspond to the sampled genes.

**Fig 1:**
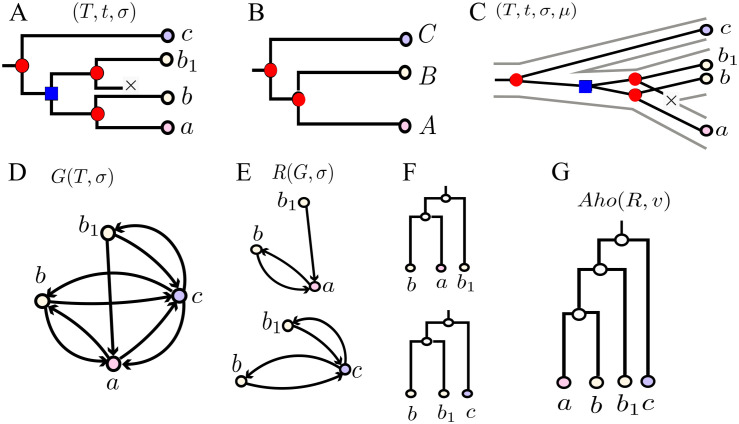
Representation of evolutionary histories and gene tree reconstruction. **A)** Event-labeled gene tree (T,t,σ) with speciation nodes shown in circles and duplication nodes in squares. Gene-to-species assignments are given by σ(a)=A, $\sigma (b)=\sigma (b_{1})=B$, and σ(c)=C. **B)** Species tree *S*. **C)** Reconciliation map μ between the gene tree (T,t,σ) and the species tree *S*, illustrated by positioning gene tree nodes within corresponding nodes or edges of ***S*. D)** Best match graph G(T,σ) constructed from the gene tree. **E)** Informative triples R(G,σ) derived from the best match graph. **F)** Aho graph based on the triples in R(G,σ). **G)** Tree reconstructed by the BUILD algorithm from the set of informative triples.

Genes evolve within genomes, i.e., species. We denote by σ:L(T)→L(S) the map that assigns each leaf in the gene tree to the species in which it resides. Moreover, we can assign evolutionary events or mechanisms to the nodes of a gene tree *T* that act on genes through evolution. Specifically, the map t:V(T)→{•,◻,×} classifies nodes in the gene tree based on evolutionary events: t(x)=• for speciation, t(x)=◻ for duplication, and t(x)=× for gene loss (see [37,38], for further details see Fig 1). Two distinct genes x,y∈L(T) are *orthologs* if $t(lca_{T}(x,y))=•$. Conversely, they are *paralogs* if $t(lca_{T}(x,y))=◻$. The inference of such evolutionary events can be approached in two ways. On the one hand, methods based on reconciling the gene tree with a known species tree are used [31,32,35,43]. On the other hand, tree-free methods enable the inference of orthologous and paralogous gene pairs without requiring knowledge of the gene tree or species tree, see [26] for an overview.

Following definitions 1, 7, and 4 of [26,37], and [8] correspondingly, an *evolutionary scenario* (S,T,t,σ,μ) consists of a species tree *S*, a labeled gene tree (T,t,σ) and a *reconciliation map* between *S* and *T*, that is, a map μ:V(T)→V(S)∪E(S) that satisfies:


(U1) Gene Constraint. If t(x)=⊙, then μ(x)=σ(x)∈L(S)(U2) Speciation Constraint. If t(x)=•, then μ(x)=lcaS(σ(CT(x)))∈V0(S), and μ(y0)∥Sμ(y1) for any two distinct children y0,y1∈childT(x)(U3) Duplication Constraint.If t(x)=◻, then μ(x)=e for some edge e∈E(S)(U4) Ancestor Constraint.If x≺Ty, then μ(x)⪯Sμ(y)

The existence of a reconciliation map between a labeled gene tree (T,t,σ) and a species tree is characterized by the compatibility of particular triples displayed in *T*. To be more precise, let $ℜ(T)=\{r\in R(T)|t(lca_{T}(L(r)))=•\text{ and }|\sigma (L(r))|=3\}$ be the set of all triples *ab*|*c* that are rooted at a speciation event, where the species σ(a), σ(b), and σ(c) are pairwise distinct. Given a triple ab|c∈ℜ(T), the corresponding *color triple* is σ(ab|c)=σ(a)σ(b)|σ(c). Finally, let 𝔖(T,t,σ)={σ(r)∣r∈ℜ(T)} be the set of *color triples* of the gene tree. The following result has been proven in [37, Thm 8] and later generalized in [38, Thm 5.4].

**Theorem 1.** *For a given labeled gene tree* (T,t,σ)*, there exists an evolutionary scenario* (S,T,t,σ,μ) *if and only if* 𝔖(T,t,σ) *is compatible. In this case, every species tree S that displays all triples in* 𝔖(T,t,σ) *can be reconciled with* (T,t,σ)*.*

In our framework, neither *T*, *t* nor *S* are given and our aim is to infer evolutionary scenarios for a given set of extant genes that solely relies on best hits, best matches and related concepts that are made more precise below.

### Best match graphs and gene trees

In what follows, we are interested in genes *y* that are the evolutionary “most closely” related ones to a given gene *x*. This concept is formalized through the notion of best matches in a given gene tree (T,σ). A gene *y* is a *best match* for a gene *x* in *T* if *x* and *y* reside in distinct species and $lca_{T}(x,y)⪯lca_{T}(x,y^{\prime })$ for all genes $y^{\prime }$ in the species σ(y). Given a gene tree (T,σ), we define the *best match graph (BMG)* G(T,σ)=(V,E) as a directed graph with node set *V* = *L*(*T*) and a directed edge xy∈E if *y* is a best match for *x* in (T,σ). Given an arbitrary colored digraph *G* and a tree *T* with *L*(*T*)=*V*(*G*), we say *T explains G* if G=G(T,σ).

**Fig 2:**
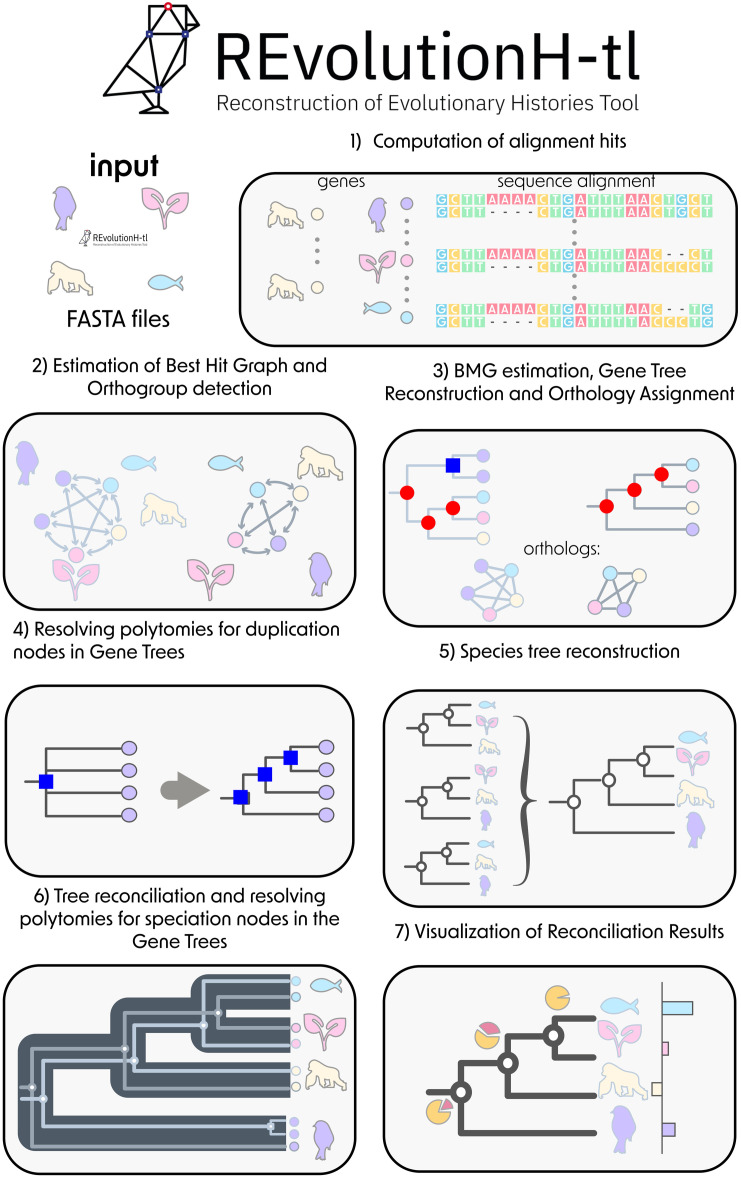
Conceptual overview of REvolutionH-tl. (1) DNA and protein alignments are computed using BLAST and Diamond. (2) BMGs are built from best hits; each connected component defines an orthogroup. (3) Gene trees are inferred per BMG, with duplication and speciation events assigned. Orthologs are identified when their last common ancestor is a speciation node. (4) Polytomic duplication nodes are resolved using a modified Neighbor-Joining algorithm. (5) A species tree is reconstructed from color triples derived from gene trees. (6) Gene trees are reconciled with the species tree to infer losses, duplications, and to refine duplication polytomies. (7) Visualizations summarize reconciliations and report statistics on orthologs and orthogroups. Species figures were generated using Canva Pro.

The structure of BMGs has been extensively studied in recent years [8,22,24,44]. In particular, as shown by Schaller et al. [23], best matches and their “symmetrized” versions paved the way to infer orthologs decreasing false-positive assignments, provided that the evolutionary history is not distorted by so-called horizontal gene transfer. Geiß et al. [8], showed that, for evolutionary scenarios that involve only speciations, gene duplications, and gene losses, there are no false-negative orthology assignments in the true bidirectional best matches. This is the case because no gene in species *A* can be more closely related to a gene in *B* than gene pairs that arose from the speciation event separating *A* and *B*. By the latter arguments, if species *B* contains an ortholog *z* of *x*, then *z* is guaranteed to be a (bidirectional) best match of *x*, assuming no horizontal gene transfer. However, not every bidirectional best matches *x* and *y* are orthologs; if an ortholog of *x* has been lost in *B*, *x* will still have a best match in *B*, provided that *B* contains any homolog of *x*, see, e.g., [23, Fig 2]. For further discussions and, in particular, a complete characterization of incorrect orthology assignments in best match graphs see [23].

A key property of best match graphs is that they encode information about gene tree topology in the so called informative triples; for any directed graph *G* and a node-coloring map σ:V(G)→M, we say that ℛ(G,σ) is the set of *informative triples* $r=xy|y^{\prime }$ with $\sigma (x)\neq \sigma (y)=\sigma (y^{\prime })$ such that xy∈E(G), $xy^{\prime }\notin E(G)$. Furthermore, if the gene tree is assumed to be binary, ℛ(G,σ) also includes those triples $yy^{\prime }|x$ whenever $xy,xy^{\prime }\in E(G)$. The following theorem was proved in [44, Thm 1] and provides a theoretical strategy to go from a data-inferred BMG, to a gene tree explaining the graph.

**Theorem 2.** *A colored digraph* (G,σ) *is a BMG if and only if the set of triples* ℛ(G,σ) *is compatible and G is explained by* T=Aho(ℛ(G,σ))*, i.e.,* (G,σ)=G(T,σ)*.*

This result allows us to first, identify if a colored digraph *G* is a BMG. In the affirmative case, we can reconstruct a gene tree by running the BUILD algorithm with the informative triples ℛ(G,σ), and in the negative case, the Aho-graph is helpful to define an heuristic for editing *G* into a BMG with minimal editions, as proposed in [24] and made more precise in the subsequent sections.

### REvolutionH-tl Methodology

The workflow of REvolutionH-tl is divided into the following 7 steps:

(1) *Computation of Alignment Hits*
(2) *Estimation of Best Hit Graph and Orthogroup Detection*
(3) *BMG Estimation, Gene Tree Reconstruction and Orthology Assignment*
(4) *Resolving Polytomies for Duplication Nodes in the Gene Trees*
(5) *Species Tree Reconstruction*
(6) *Tree Reconciliation and Resolving Polytomies for Speciation Nodes in the Gene Trees*
(7) *Visualization of Reconciliation Results*.

This methodology is illustrated in Fig 2.

### Computation of alignment hits

The aim of this step is to compute sequence similarity between genes of different species. The input is a collection of FASTA files, each of them corresponding to one species and containing a list of sequences. Alignments are computed using Diamond [10,11] for amino acid sequences or BLAST [45,46] for DNA sequences. As output, REvolutionH-tl generates a directory containing the alignment hits for each pair of species.

### Estimation of best hit graph and orthogroup detection

REvolutionH-tl takes the alignment hits between genes from different species and calculates a normalized bit score to approximate evolutionary relatedness. Following the method in [28], a gene *y* in species *B* is considered a best hit of *x* if its bit score falls within an adaptive threshold relative to the top-scoring hit. In particular, we consider nearly co-optimal best hits by considering for a given gene *x* in species σ(x) all those genes y∈B having a normalized bit score not less than *f* times the score of the most similar gene in *B* [28]; by default, we set *f* = 0.95. This process produces a directed *best hit graph* (H,σ), which reflects the sequence similarity relationships.

**Fig 3:**
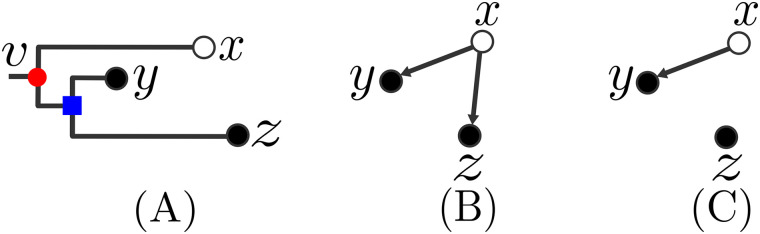
Best matches and best hits. **(A)** A gene tree with three genes *x*, *y*, and *z* from two species (represented in black and white), rooted at node ***v*.** The least common ancestor of *x* relative to *y* and *z* is ***v*.** The similarity score (measured as weighted tree distance) between *x* and *y* is higher than that between *x* and *z*, indicating that the Strict Molecular Clock Hypothesis does not hold in this case. **(B)** Genes *y* and *z*, both from the black species, are best matches of ***x*. (C)** Gene *y* is the best hit of *x*, whereas *z*, though a best match, is not considered a best hit.

The connected components of a best hit graph are interpreted as *orthogroups*, and each of the orthogroups will be represented by a single gene tree. In practice, merging two connected components is likely to introduce false positive homology relations. From the theoretical side, if we consider the tree of a disconnected best match graph (see for example [47, Fig 3]), each connected component is contained in a different sub-tree rooted at a child of the root and all the species are represented in the members of the connected component [47, Thm 1]. Thus, given the orthogroup *A* and a gene a∈A, all the orthologs of *a* are contained in *A*. We expect the best hit graphs to resemble this property, because best hits are a data-driven approximation for best matches. Furthermore, if all the orthologs of *x* in a species are lost, another homolog *y* in such species will be part of the orthogroup if it exists. Note that this does not implies *y* is ortholog of any member of *A*.

Traditionally, orthogroups are defined as sets of genes descended from a single gene in the last common ancestor of the species under consideration, e.g., [30]. This definition implies that for an orthogroup *A*, the last common ancestor of *A* in the gene tree corresponds to a speciation event (in the absence of HGT). Our definition is more relaxed, while still providing essentially the same information about the evolutionary relations between genes, as explained in the following section. In particular, a duplication appears as last common ancestor of a connected component of a best match graph if there is a pair of species in which no orthologs have survived and thus ancestral paralogs are the only surviving homologs. Using this information, it is easy to recover ‘classical orthogroups’ as a subset of connected components of best match graphs.

The output of this step includes two tsv files: one listing the best hit gene pairs, and the other containing the identified orthogroups.

### BMG estimation, gene tree reconstruction and orthology assignment

For a given gene *x* in species *A*, a gene *y* in species *B* is defined as a *best match* if it is evolutionarily the most closely related gene to *x* among all genes in species *B* [47]. Note that a gene may have more than one best match and, moreover, it is important to distinguish between best hits and true best matches, as illustrated in Fig 3.

We follow the approach described in [21,24] to approximate best matches using the best hit graph. Recall that an orthogroup corresponds to a connected component of the best hit graph (H,σ). We examine each orthogroup individually, assuming that the orthogroup in question corresponds to the connected component (G,σ) of (H,σ). For each orthogroup, we extract the set ℛ≔ℛ(G,σ) of informative triples from (G,σ). If the best hit graph coincides with the true BMG, then ℛ(G,σ) must be compatible, and we can apply the BUILD algorithm [39,42] to construct a gene tree T=Aho(ℛ) for the orthogroup. In this case, Theorem 2 guarantees that the BMG (G,σ) coincides with the BMG derived from (T,σ). However, since the best hit graph is only an approximation of the true BMG, the resulting triple set ℛ might not be compatible. To address this, following [24,48], REvolutionH-tl applies a heuristic to identify a maximum subset of compatible triples, denoted $\hat{ℛ}$. This heuristic modifies the behavior of BUILD by enforcing a partition of the auxiliary Aho graph whenever it remains connected, thereby ensuring progress even in the presence of conflicts or inconsistencies. The gene tree (T,σ) is then reconstructed from $\hat{ℛ}$ using the BUILD algorithm, and the graph G(T,σ) serves as the *estimated* BMG of the corresponding orthogroup. Note that *T* is not necessarily fully resolved.

Up to this point, we have reconstructed a gene tree (T,σ) for each orthogroup, which we now use to infer orthologs within the orthogroup. To this end, we must first assign evolutionary events, such as speciations and duplications, to the internal nodes of (T,σ). Following [8,49], an internal node *v* of *T* is labeled as a *speciation* event if the sets of species in its child subtrees are pairwise disjoint, and as a *duplication* event otherwise. This yields an event-labeled gene tree (T,t,σ), where *t* assigns to each internal node *v* of *T* its corresponding event, that is, *t*(*v*) is either a speciation or a duplication.

The gene tree *T* obtained by the BUILD algorithm is not expected to be fully resolved. Here, the event labeling we use introduces the possibility of wrongly labeling polytomies as duplication events. We address this issue using the approach proposed in [23,50] by constructing an *augmented tree* where some polytomies marked as duplication are resolved into an speciation event followed by duplications. This approach assumes the least resolved gene tree explaining the best match graph while minimizing gene duplication and loss.

From the event-labeled tree, we can readily obtain orthologous gene pairs that are identified as those whose least common ancestor in *T* is labeled as a speciation event. A key strength of this relation-based approach is its robustness, i.e., we do not require fully resolved gene trees since orthology assignments are determined by the *discriminating tree* [51], which is obtained by collapsing consecutive internal nodes with the same event label. Thus, even a partially resolved gene tree with a few incorrect branches can yield accurate orthology predictions, as long as the overall structure captures the correct evolutionary splits.

The output of this step are three files in tsv format: (1) a list of inferred gene trees in NHX format, each associated with its orthogroup ID; (2) a list of best matches recomputed from (T,t,σ); and (3) a list of orthologous gene pairs derived from the event-labeled tree.

### Resolving polytomies for duplication nodes in the gene trees

Gene trees (T,t,σ) produced in the previous step are generally not fully binary. We consider two different ways to resolve polytomies based on the event-labeling *t*(*x*) of a node *x* in *T*. To resolve polytomies labeled as speciation events, we leverage *t*he reconciliation map and the topology of the species tree as outlined in more detail in Section *Tree Reconciliation and Resolving Polytomies for Speciation Nodes in the Gene Trees*.

For now we focus on duplication nodes. For polytomies labeled as duplication events, we apply a distance-based clustering strategy that incrementally builds a binary subtree using a neighbor joining (NJ) criterion, as described in Algorithm 1. The distances used for NJ are score-distances, cf. [52].

Let x∈V(T) be a duplication node in a gene tree *T*, with children $child(x)=\{y_{1},y_{2},...,y_{k}\}$, where k≥3. The goal is to replace the unresolved polytomy at *x* with a fully resolved binary subtree $T^{\prime }$ that has leaf-set $\left\{y_{1},y_{2},...,y_{k}\right\}$.

We begin by initializing an unrooted star-shaped tree $T^{\prime }$, whose leaf set is $L(T^{\prime })=\{y_{1},y_{2},...,y_{k}\}$, and a central internal node $x^{\prime }$. We also define a set of clusters $C=\{C_{1},C_{2},...,C_{k}\}$, where each cluster $C_{i}$ corresponds to the leaves of the subtree $T(y_{i})$. For each pair $y_{i},y_{j}\in child(x)$, we compute a pairwise distance $d(y_{i},y_{j})$ as the average of all distances $\delta (z_{i},z_{j})$ between gene pairs $(z_{i},z_{j})\in C_{i}\times C_{j}$. The distances δ are derived from sequence similarity scores obtained during the initial comparison step using DIAMOND or BLAST. Even though the minimum distance would be a theoretically better estimate of the divergence between the clusters, the stochastic nature of the minimum distances may introduce a strong bias in the estimations.

With the star tree $T^{\prime }$ and the distance matrix *d* in place, the algorithm proceeds iteratively. At each step, the pair $y_{i},y_{j}\in N(x^{\prime })$ minimizing the NJ criterion is selected. These nodes are merged into a new internal node $y_{ij}$: the edges $x^{\prime }y_{i}$ and $x^{\prime }y_{j}$ are removed, and new edges $x^{\prime }y_{ij}$, $y_{ij}y_{i}$, and $y_{ij}y_{j}$ are added. The distance matrix is updated using standard NJ rules to reflect the distances between $y_{ij}$ and the remaining neighbors of $x^{\prime }$. This iterative process continues until $T^{\prime }$ is fully resolved. A root is then added to $T^{\prime }$ using the midpoint rooting method [53]. We use the Biopython implementation of NJ and midpoint rooting algorithms [54]. The resulting binary subtree $T^{\prime }$ is then grafted back into the original gene tree *T* as follows. Let $x^{\prime }$ be the parent of *x* in *T* and $Y_{i}=child_{T}(y_{i})$ for all $y_{i}\in child_{T}(x)$, we update *T* by deleting the nodes $x,y_{1},y_{2},...$ together with the adjacent edges, and adding the edge $x^{\prime }\rho_{T^{\prime }}$ connecting $x^{\prime }$ with the root of $T^{\prime }$ as well as the edges $y_{i}y_{i}^{\prime }$ for all $y_{i}\in L(T^{\prime })$ and $y_{i}^{\prime }\in Y_{i}$. Because the method relies solely on distance information and does not impose external biological constraints, it is particularly suitable for resolving duplication nodes in cases where topological refinement is needed independently of additional annotations.

**Algorithm 1: Resolve-Duplication-Polytomy**



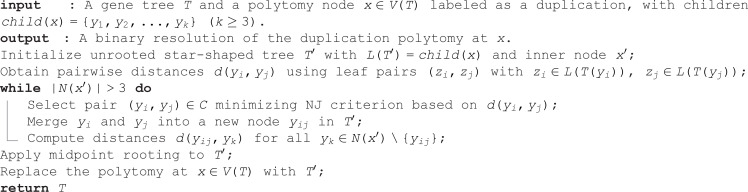



### Species tree reconstruction

Event-labeled gene trees (T,t,σ) contain implicit information about the underlying species tree *S* in the form of gene triples of type *uv*| *w*, where *u*, *v*, and *w* are genes from three different species. If their least common ancestor *lca*(*u*, *w*) = *lca*(*v*, *w*) is labeled as a speciation event, then the corresponding color triple σ(u)σ(v)|σ(w) is displayed by the species tree [37,38,55]. Let us denote with 𝔖≔𝔖(T,t,σ) the collection of all such color triples derived from (T,t,σ).

In the absence of noise (e.g., measurement errors or horizontal gene transfer) the set 𝔖 is compatible, and (T,t,σ) is always reconcilable with the species tree S=Aho(𝔖) (cf. Theorem 1). Note that *S* is not necessarily binary; however, any refinement of *S* yields a tree $S^{\prime }$ with which (T,t,σ) remains reconcilable [37,38]. Because we follow a heuristic approach, we cannot guarantee that 𝔖 is always compatible. Analogous to the treatment of gene triples, we therefore follow [24,48], where REvolutionH-tl applies a heuristic to identify a maximum subset of compatible triples, denoted by $\hat{𝔖}$. The species tree is then defined as $S=\text{Aho}(\hat{𝔖})$.

The output of this step is a single file containing the inferred species tree in NHX format.

### Tree reconciliation and resolving polytomies for speciation nodes in the gene trees

Recall that the species tree *S* for an event-labeled gene tree (T,t,σ) is obtained by heuristically determining a maximum compatible subset $\hat{𝔖}$ of 𝔖≔𝔖(T,t,σ) and setting $S=\text{Aho}(\hat{𝔖})$. Consequently, it may occur that a color triple σ(ab|c)∈𝔖, corresponding to a gene triple *ab*|*c* where $lca_{T}(a,b,c)$ is a speciation node, is not displayed by the species tree *S*. In this case, (T,t,σ) is not reconcilable with *S*, and we therefore further modify (T,t,σ) as follows.

Let $R_{S}$ denote the set of triples displayed by *S*. By construction, $\hat{𝔖}\subseteq R_{S}$. We define $R_{C}≔R_{S}\cap 𝔖$ as the set of colored triples in (T,t,σ) that are displayed by *S*, and $R_{I}≔R_{S}⧵𝔖$ as the set of inconsistent colored triples.

For each leaf *x* of *T*, we count the number of triples ab|c∈R(T) in which x∈{a,b,c} and for which $\sigma (ab|c)\in R_{I}$, and denote this count by $w_{x}^{I}$. Analogously, let $w_{x}^{C}$ denote the number of such triples with $\sigma (ab|c)\in R_{C}$. We then remove from *T* the leaf *x* that maximizes $w_{x}^{I}$ and, among all such leaves, minimizes $w_{x}^{C}$. After removing *x*, all triples $\sigma (ab|c)\in R_{I}$ involving *x* are deleted. This procedure is repeated until $R_{I}$ becomes empty (see Algorithm 2). The result is a pruned gene tree $\left(T^{\prime },t^{\prime },\sigma^{\prime }\right)$, where $t^{\prime }$ and $\sigma^{\prime }$ are inherited from *t* and σ, respectively, retaining the original node labels and leaf colors.

This pruning process of (T,t,σ) ensures that the remaining triple set $R_{C}$ is a subset of the colored triple set $\hat{𝔖}$, and thus, by Theorem 1, the resulting tree $\left(T^{\prime },t^{\prime },\sigma^{\prime }\right)$ is reconcilable with the species tree *S*.

**Algorithm 2: Prune-L: Correction of inconsistent gene trees**



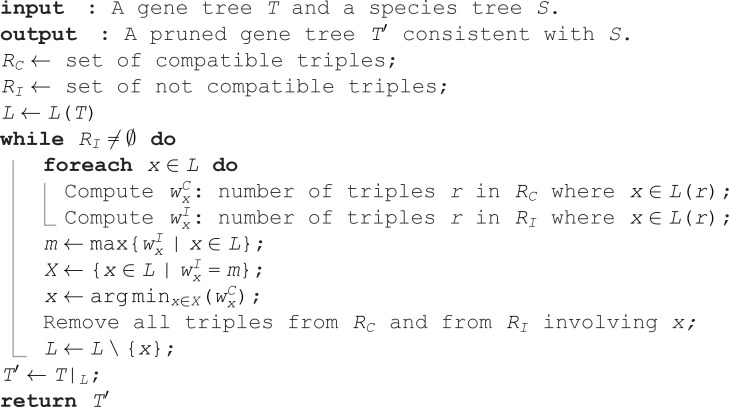



Once the gene tree has been pruned and is consistent with the species tree, reconciliation is performed by mapping each node of the gene tree to a node or edge in the species tree according to its evolutionary label (see the Constraints (U1)-(U4)).

For simplicity, we write $\left(T,t,\sigma )≔(T^{\prime },t^{\prime },\sigma^{\prime }\right)$. The pruned gene tree (T,t,σ) may still contain unresolved polytomies. As outlined above, duplication nodes are resolved using the modified NJ algorithm. To resolve non-binary speciation nodes in (T,t,σ), we leverage the reconciliation map μ and the topology of the species tree *S*. To be more precise, let *x* be a node in *T* such that *t*(*x*) is a speciation event, and let $child(x)=\{y_{1},y_{2},...,y_{k}\}$ denote its set of children, with k≥3. Each child $y_{i}$ is mapped by μ to an edge or vertex of *S*. By Condition (U2), for any two distinct children $y,y^{\prime }\in child(x)$ the images μ(y) and $\mu (y^{\prime })$ are incomparable in *S*. This property allows us *t*o define the subtree $S^{\prime }$ of *S* induced by μ(x) and all vertices along the unique paths from μ(x) to μ(y) for all y∈child(x) (suppressing vertices of in- and out-degree one). We then replace the star-shaped subtree of *T* induced by *x* and its children with the tree $S^{\prime }$. If the species tree *S* is binary (for instance, when provided as input by the user), this procedure - together with the resolution of duplication nodes - ensures that the resulting gene tree is fully binary.

Gene loss events are inferred during reconciliation by examining speciation nodes in the gene tree. When a speciation node *u* in the gene tree is mapped to an internal node *v* in the species tree, we verify whether each child of *v* is represented among the descendants of *u*. If this is not the case, gene loss events are inferred accordingly.

The input for this step consists of a list of gene trees and a species tree, both in NHX format. The user may provide a species tree, or it can be inferred automatically by REvolutionH-tl as described in the previous section. The output is a tsv file containing evolutionary scenarios. Each entry includes the orthogroup identifier, the reconciled gene tree in NHX format, and a reconciliation map that associates each node in the gene tree with its corresponding node or edge in the species tree. Since only duplication nodes are mapped to edges, we report the child node *v* of the edge *uv* in *S* as the reconciliation target for a duplication node. As a result, the reconciliation map is presented as a list of node pairs. The reconciled gene trees produced by this step explicitly annotate evolutionary events, including gene duplications and losses.

### Visualization of reconciliation results

We provide dedicated commands for visualizing the reconciliation results. The command revolutionhtl.plot_reconciliation allows users to display the evolutionary scenario of a specific orthogroup by embedding the gene tree within the species tree. This visualization facilitates intuitive interpretation of the gene-species mapping and the associated evolutionary events. An example is shown in Fig 7.

For a broader summary across multiple gene families, the command revolutionhtl.plot_summary generates two comprehensive diagrams: (i) the *reconciliation tree*, and (ii) a diagram illustrating the *relative change in gene content* across clades.

**Fig 4:**
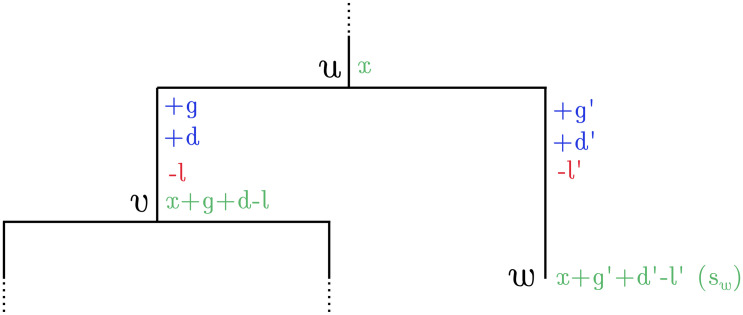
Explaining change in gene content. We show two inner nodes and one leaf of a species tree, labeled as *u*, *v*, and *w* respectively. Additionally, we add some integer variables explaining the evolution of gene content for these three elements. For the inner nodes *u* and *v*, the green numbers correspond to genes present in a species just before the speciation event, for example, node *u* has *x* genes while *v* has x+g+d−l. Numbers along the branch *uv* display gene acquisitions and losses in color blue and red correspondingly; *g* is the number of gained *g*enes (corresponding to the number of gene trees whose root maps to *v*, in other words, this gene family specific for *v* and descendants) while *d* stands for the number of duplicated genes, and the red number *l* corresponds to gene loss. The gene content of *l*eaf *w* is explained similarly, we just add an extra variable in parenthesis, $s_{w}$, which shows the number of species-specific genes, also known as singletons.

**Fig 5:**
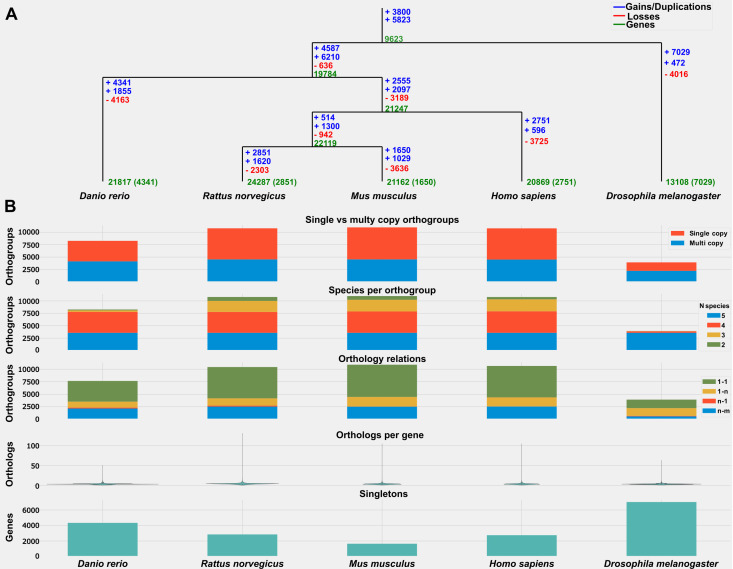
Genome complexity evolution for the Phylome05 dataset. The species tree shown at the top of the figure defines the left-to-right order of species along the x-axis: *Danio rerio*, *Rattus norvegicus*, *Mus musculus*, *Homo sapiens*, and *Drosophila melanogaster*. Each column in panels (B–F) corresponds to one of these species, as ordered by the tree, and displays species-specific data. For example, the column below *Danio rerio* exclusively reports statistics derived from orthogroups that contain at least one gene from this species. **(A)** Evolution of genome complexity, following the notation introduced in Fig 4. Gene counts per species are shown in green, while blue and red indicate gene gains/duplications and gene losses, respectively. **(B)** Proportion of orthogroups consisting of single-copy genes versus those containing paralogs. **(C)** Distribution of orthogroups based on the number of species represented within each group. **(D)** Classification of orthology relationships for genes from the species associated with the current column. Relationships are categorized as one-to-one, one-to-many, many-to-one, or many-to-many across species. **(E)** Number of orthologs per gene for the species indicated by the column. **(F)** Total count of species-specific genes. Font sizes in the figure were manually adjusted to enhance readability.

The reconciliation tree provides an overview of gene content evolution across the species tree. It reports counts of inherited genes, duplicated genes, clade-specific gains, and species-specific genes, as illustrated in Fig 4. Additionally, it summarizes orthogroup statistics, including the number of single-copy orthogroups, the average number of species per orthogroup, classifications of orthology relationships, and the total number of single-copy genes. A representative example is shown in Fig 5.

**Fig 6:**
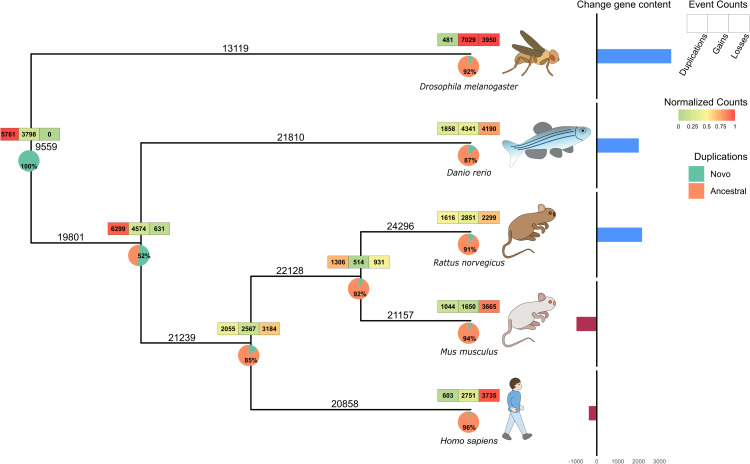
Visualization of relative change in gene content for the Phylome05 dataset using REvolutionH-tl. Summary of key evolutionary events across the phylogeny, including gene duplications, gains, and losses. A heatmap along the branches encodes normalized event values, where red indicates below-average and green indicates above-average frequencies. At each internal node, pie charts represent the proportion of *de novo* versus ancestral gene duplications. To the right of the tree, bar charts depict changes in gene content across species: blue bars indicate larger gene gains, while wine-colored bars correspond to larger gene losses. Visual representations of the analyzed species were added manually to enhance interpretability.

The second diagram highlights clades undergoing the most significant shifts in gene content. It reports the percentage of ancestral versus *de novo* duplications and applies min-max normalization to the metrics reported in Fig 4. This visualization enables quick identification of clades with particularly dynamic evolutionary histories. An example is shown in Fig 6.

All visualizations are generated using custom R scripts (v4.4.0) integrated into the REvolutionH-tl framework. The layout and design were inspired by visualization strategies proposed in [56].

### Benchmarking

To show the correctness of our approach, we run the REvolutionH-tl workflow to predict (i) orthogroups, (ii) orthology, (iii) gene trees, (iv) species trees, and (v) tree reconciliation. Then, we contrasted these predictions against those made by Proteinortho, OrthoFinder, RANGER-DTL, RAxML, GeneRax, and ASTRAL-Pro. Table 1 illustrates how we compare the outputs of revolutionhtl and the other tools.

**Table 1:** Input and output of the benchmarking tools. Rows of this table corresponds to the different tools used for the benchmarking analysis. On the other hand, columns of the table show input/output files. The checkmark (✓) indicates the outputs that we keep for each took, similarly, the letter *I* indicates the input files.

	Molecular Model	MSA	genes/proteins	Orthogroups	Orthology	Gene Trees	Species Trees	Reconciliation
**REvolutionH-tl**			I	✓	✓	✓	✓	✓
**Proteinortho**			I	✓	✓			
**OrthoFinder**			I	✓	✓			
**RAxML**	I	I				✓		
**GeneRax**	I	I					I	✓
**ASTRAL-Pro**						I	✓	
**RANGER-DTL**						I	I	✓

#### Synthetic dataset.

We use SaGePhy [57] to simulate evolutionary histories under three main parameters: the number of species N∈{5,10,15,...,50}, duplication rate *d*, and loss rate *l*. The parameters *d* and *l* are independent*l*y and uniform*l*y sampled from the set {i/10∣i∈ℕ, 0≤i≤10}, resulting in a total of 1,210 distinct parameter combinations (*N*, *d*, *l*).

For each parameter triple, we simulate five independent evolutionary scenarios on a species tree *S*, resulting in five gene trees that evolve within *S*. The simulated trees include branch lengths, which reflect evolutionary rates under the assumption of a constant mutation rate across all genes. Protein sequences are then generated based on these trees using the *LG substitution model* [58], one of the models provided by SaGePhy.

To construct genome-like datasets, we extract the simulated protein sequences and generate a set $F_{N}$ of FASTA files, one per species. Each FASTA file represents the complete proteome of a simulated species. We refer to the resulting dataset of simulated genomes and evolutionary histories as SaGePhy5–50.

This synthetic dataset provides a controlled ground truth for evaluating inference tools. Because the evolutionary scenarios are fully known, we can directly extract the *true* orthogroups by taking the set of leaves in each gene tree, and orthologs by considering pairs of leaves whose last common ancestor is a speciation event.

Performance evaluation is then conducted by comparing these ground truth annotations to the predictions produced by each tool.

#### Empirical genomic datasets.

To demonstrate the application of our tool on real biological data, we selected five species from the (PhyId 500) [QfO] Reference Model Species Metaphylome dataset available in the PhylomeDB database [49]. The selected species are: *Homo sapiens*, *Mus musculus*, *Rattus norvegicus*, *Danio rerio*, and *Drosophila melanogaster*. For each species, we obtained FASTA files containing protein sequences. To ensure a consistent representation of gene loci, we excluded protein variants and isoforms, retaining only a single representative sequence per gene. It is important to note that, because we use proteome data, the sequences in these FASTA files correspond exclusively to protein-coding genes. We refer to this dataset as Phylome05, and use it to evaluate the behavior and outputs of REvolutionH-tl   on high-quality, curated genomic data.

To evaluate scalability, we used the dataset prt10–250 from the benchmarking study of Proteinortho6 [12]. We also compare running times on this dataset for OMA, Proteinortho, and SonicParanoid. The prt10–250 dataset comprises three collections of randomly selected bacterial genomes containing 10, 100, and 250 species, respectively. These collections were used as input for REvolutionH-tl   to assess performance as the number of species increases.

#### Correctness of orthogroup identification.

Accurately identifying homologous genes is a fundamental step in evolutionary analysis. To evaluate the correctness of orthogroup predictions on the synthetic dataset, we compare the results produced by REvolutionH-tl, ProteinOrtho, and OrthoFinder against the ground-truth orthogroups generated using SaGePhy.

To assess performance, we classify each ground-truth orthogroup according to the relationship between its true members and the predicted orthogroups, as illustrated in Fig 8. A ground-truth orthogroup *X* is considered *fully recovered* if there exists a predicted orthogroup $X^{\prime }$ such that $X=X^{\prime }$. If no such match exists, the prediction is deemed incorrect.

Incorrect predictions are further categorized into two error types:

- Split orthogroups: A true orthogroup *X* is considered split if the predicted orthogroup is a proper subset, i.e., $X^{\prime }\subset X$. This indicates that the tool failed to recover all members of the group in a single cluster.
- Merged orthogroups: Two or more true orthogroups (e.g., *X*_0_ and *X*_1_) are considered merged if a predicted orthogroup $X^{\prime }$ contains elements from both, i.e., $X^{\prime }\cap X_{0}\neq \emptyset$ and $X^{\prime }\cap X_{1}\neq \emptyset$. This suggests an over-clustering of unrelated gene sets.

For each tool, we report the percentage of correctly recovered, split, and merged orthogroups to quantify its performance in orthogroup identification.

#### Standard performance metrics.

To evaluate the performance of tools for orthology prediction and gene tree inference, each individual prediction is classified as a *true positive* (TP), *false positive* (FP), *true negative* (TN), or *false negative* (FN). These predictions may correspond to orthology relationships or resolved triplets in a gene or species tree.

Once the predictions have been categorized, we compute the standard performance metrics as follows. Precision is defined as $\frac{TP}{TP+FP}$, measuring the proportion of correct positive predictions. Recall is given by $\frac{TP}{TP+FN}$, representing the proportion of actual positives that are correctly identified. The false positive rate (FPR) is computed as $\frac{FP}{FP+TN}$, indicating the proportion of negatives incorrectly classified as positives. Finally, accuracy is calculated as $\frac{TP+TN}{TP+FP+TN+FN}$, reflecting the overall proportion of correct predictions.

For each orthogroup *X*, we calculate its performance value $A_{X}$ according to each of the metrics described. To assess trends under specific evolutionary conditions, orthogroups are grouped into collections based on shared simulation parameters, such as fixed duplication and loss rates. For a collection of orthogroups $Y=\{X_{0},X_{1},...\}$, the average performance for a given metric is computed as:


$$\begin{array}{c} A_{Y}=\frac{1}{\left|Y\right|}\sum_{X\in Y}A_{X}. \end{array}$$

This aggregated evaluation enables a consistent and robust comparison of inference tools across diverse evolutionary scenarios.

#### Performance for orthology inference.

We evaluate the accuracy of orthology prediction at the level of individual orthogroups. Let *X* be a true orthogroup, defined as a set of genes. The set of true orthology relations within *X* is denoted by ΘX⊆(X2).

Let IX⊆{uv∈(X′2)∣u∈X or v∈X} be the set of orthology relations inferred by a method, where $X\subseteq X^{\prime }$ and $X^{\prime }$ includes genes that do not belong to the true orthogroup *X* but are *incorrectly* predicted to be orthologs to genes in *X*; we collect such ‘crossing edges’ in the set $I_{X}^{cross}=\{uv\in I_{X}|u\in X,\;v\in X^{\prime }⧵X\}$. This formulation allows the consideration of orthogroups with both over-clustering and under-clustering errors.

We define the universe $U_{X}$ of possible relations for the orthogroup *X* as the set containing all pairs of genes in *X* and the inferred orthology relations where one gene belongs to *X* and the other lies outside *X*, thus |UX|=(|X|2)+|IXcross|.

We classify the outcomes of the predictions by computing the number of true positives as $tp=|I_{X}\cap \Theta_{X}|$, the number of false positives as $fp=|I_{X}⧵\Theta_{X}|$, the number of false negatives as $fn=|\Theta_{X}⧵I_{X}|$, and the number of true negatives as $tn=|U_{X}|-tp-fp-fn$. Thus accounting for all pairwise gene combinations, including those within the orthogroup, between orthogroup and external genes.

Based on these quantities, we compute standard performance metrics such as precision, recall, accuracy, and false positive rate.

#### Performance for tree reconstruction.

To assess the accuracy of inferred phylogenies, we compare each reconstructed tree $T^{\prime }$ with its corresponding ground-truth tree *T* by evaluating the sets of rooted triples they induce. A rooted triple represents the resolved relationship among a triplet of taxa in the tree.

Let *R*(*T*) and $R(T^{\prime })$ be the sets of rooted triples derived from the true and inferred trees, respectively. From these, we calculate the number of true positives as $\left|R(T)\cap R(T^{\prime })\right|$, the number of false positives as $\left|R(T^{\prime })⧵R(T)\right|$, and the number of false negatives as $\left|R(T)⧵R(T^{\prime })\right|$. The number of true negatives is computed as 2(n3)−TP−FP−FN, where *n* denotes the number of taxa.

These values form the basis for standard performance metrics, allowing us to evaluate the quality of tree reconstruction in terms of precision, recall, and false positive rate, relative to the resolved triplets in the true topology.

#### Reconciliation distance.

To assess the quality of gene tree reconstructions, we reconciled the event-labeled gene trees inferred by all methods with the true species tree. This enabled us to apply the *PLR* dissimilarity measure [59], which estimates the confidence in reconciliation results by comparing evolutionary scenarios.

Given two evolutionary scenarios, ${𝒢}_{1}=(S,T_{1},t_{1},\sigma_{1},\mu_{1})$ and ${𝒢}_{2}=(S,T_{2},t_{2},\sigma_{2},\mu_{2})$, the *PLR* dissimilarity is defined as:


$$\begin{array}{ccc} d_{plr}({𝒢}_{1},{𝒢}_{2}) & = & d_{asym}({𝒢}_{1},{𝒢}_{2})+d_{asym}({𝒢}_{2},{𝒢}_{1}) \end{array}$$ (1)

$$\begin{array}{ccc} d_{asym}({𝒢}_{1},{𝒢}_{2}) & = & \alpha \cdot d_{path}({𝒢}_{1},{𝒢}_{2})+(1-\alpha )\cdot d_{lbl}({𝒢}_{1},{𝒢}_{2}) \end{array}$$ (2)

Equation 1 ensures symmetry, i.e., $d_{plr}({𝒢}_{1},{𝒢}_{2})=d_{plr}({𝒢}_{2},{𝒢}_{1})$, while Equation 2 shows that the dissimilarity is composed of two components: the *path* component and the *label* (*lbl*) component.

Both components rely on a node-mapping function *m*, which maps each node $x\in V(T_{1})$ to a corresponding node $y\in V(T_{2})$, defined as $y=m(x)=lca_{T_{2}}(L(T_{1}(x)))$. The *path* component measures the number of edges between $\mu_{1}(x)$ and $\mu_{2}(y)$ in the species tree *S*, capturing the topological distance between the mapped positions. The *lbl* component reflects differences in the evolutionary event labels of *x* and *y*, increasing by one if $t_{1}(x)\neq t_{2}(y)$, and zero otherwise.

In our experiments, we set α=1/n, as recommended in [59], where *n* is the number of species in the species tree.

This reconciliation-based measure allows us to simultaneously evaluate structural differences between gene trees and inconsistencies in the predicted reconciliation mappings and evolutionary events, providing a comprehensive and interpretable metric for assessing gene tree accuracy.

### Quest for orthologs benchmark

We further evaluate the precision of orthology prediction by running the gold standard benchmarking service *Quest for Orthologs* (QfO) with the dataset 2022_02, consisting of 78 curated proteomes from all domains of life [60,61].

This service offers 13 different challenges and 34 charts for measuring performance, publicly available at [62]. Since the dataset comprises true genomes sequenced from living organisms, there is no ground truth for orthologous relations or evolutionary scenarios. For this reason, the QfO pipeline offers three surrogate measurements of the correctness of orthology prediction, which are described below; further details are in [60].

(1) *Generalized species tree discordance*. This type of benchmark constructs a tree for different samples of sequences where every pair of genes is predicted to be orthologs, and thus related by speciation. Then, precision is estimated as the complement of the Robinson-Foulds (RF) distance between the estimated trees and the ground truth species tree, i.e., the smaller the distance, the higher the precision. This test is applied in 4 datasets: *Eukaryota*, *Fungi*, *Vertebrata*, and *Luca*. A non-generalized variant of this test with a restricted set of species tree topologies is applied to *Bacteria* and *Eukaryota*.
(2) *Reference gene trees*. The predicted orthology relationships are compared with those derived from reference event-labeled gene trees in the SwissTree and Treefam-A databases. Every pair of genes in a reference tree is classified as true/false positive/negative, these values are used to compute positive predictive values *PPV* = |*TP*|/(|*TP*| + |*FP*|) as a measure of precision, and true positive rate *TPR* = |*TP*|/(|*TP*| + |*FN*|) as a measure of recall. This test is applied to the gene trees from *SwissTree*, *TreeFam-A*, and *VNGC*.
(3) *Functional tests*. Annotations from UniProt-GOA, together with hierarchical EC number assignments from the ENZYME database maintained by Swiss-Prot, were used to measure functional similarity between predicted orthologs using semantic similarity. This test is applied to the annotations from UniProt–Gene Ontology Annotation (GOA) database and Enzyme Commission (EC) numbers from the ENZYME database

However, there is a bias in the QfO tool with respect to the recall metric; in benchmark types (1) and (3), recall is approximated as the number of trees that can be sampled and the number of inferred orthologs, respectively [60]. These metrics outperform those tools that produce large sets of orthology predictions, even if the correctness of those predictions is questionable [12]. Consequently, we report only the precision results for these types of benchmarks.

Among the 34 different charts for measuring orthology precision, approximately half yielded identical precision results. We therefore removed duplicates and report only 19 benchmark datasets with unique average precision. Specifically, we remove 7 tests among those using the fraction of incorrect trees as the metric and 7 among those using the RF average as the metric. Each benchmark chart in [62] corresponds to a combination of two metrics. For example, STD_Fungi in Fig 10 stands for the Species Tree Discordance benchmark in Fungi, with four tests run on this dataset. These tests are visualized in a Pareto plot (see S2 Appendix), where the y-axis measures average precision (i.e., average fraction of incorrect trees or average RF distance). Among the four STD_Fungi tests, two share the same average precision.

## Results

Our primary contribution is REvolutionH-tl, an open-source, cross-platform Python tool [63] developed for fast and accurate orthology prediction, as well as the inference and reconciliation of gene and species trees. The design and functionality of REvolutionH-tl make it particularly well-suited for large-scale comparative genomics analyses.

Since its initial release [27], we have introduced several enhancements to both the software and the accompanying benchmarking framework to further validate the correctness of our inferences. Methodological improvements include the integration of a novel module for polytomy resolution. This module complements the existing best-match strategy by enabling the binarization of polytomies labeled as duplication events, thereby improving the biological realism of the inferred gene trees.

In addition to methodological advancements, we have expanded the software’s visualization capabilities. New commands now allow for the graphical exploration of evolutionary scenarios, facilitating integrative and interpretable analyses. Furthermore, runtime performance has been significantly improved through the parallelization of key modules responsible for processing alignment hits and selecting best matches.

To evaluate the performance of REvolutionH-tl, we conducted an extended benchmarking study that includes six widely used tools for evolutionary analysis: Proteinortho, OrthoFinder, RAxML, ASTRAL-Pro, RANGER-DTL, and GeneRax. For each of these tools, we used the latest available version to ensure a fair and up-to-date comparison.

### Evolution visualization

We provide the command revolutionhtl.plot_summary for the comprehensive aggregation and visualization of the evolutionary history of multiple gene families. This functionality is exemplified in Figs 5 and 6, which illustrate the evolution of genome complexity across metazoan species included in the Phylome05 dataset. The evolutionary scenarios for this analysis were inferred directly from FASTA files using the command python -m revolutionhtl -F fastas/.

In addition, this plotting command accepts parameters that allow users to focus on a custom subset of orthogroups, enabling targeted analyses of specific gene families of interest.

Additionally, the command revolutionhtl.plot_reconciliation enables the visual inspection of complex evolutionary scenarios by embedding gene trees within the species tree context. In these visualizations, the species tree is represented as a gray pipe along which genes evolve. Gene trees are drawn in black, with bifurcations annotated to indicate evolutionary events: • marks speciation events, and ■ denotes gene duplications. Gene tree leaves are also color-coded—red for extant genes and black for inferred gene losses.

**Fig 7:**
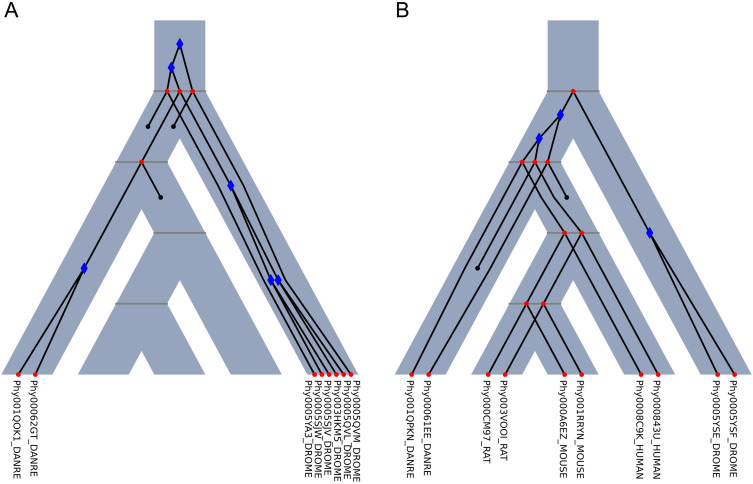
Evolutionary scenarios from the Phylome05 dataset. The species tree is represented as a gray pipe, within which the gene trees evolve. Gene trees are overlaid in black, with bifurcations annotated to indicate evolutionary events. Divergences caused by speciation are marked with •, while gene duplication events are indicated with ■. Additionally, gene tree leaves are color-coded: red denotes extant genes, whereas black signifies gene loss events.

For example, Fig 7A illustrates a gene family with multiple duplication events, both ancestral and species-specific, as well as three gene losses. This results in a family composed of only eight genes distributed across two species. In contrast, Fig 7B shows a more conserved scenario in which the gene family is retained across all sampled species. These visualizations facilitate the interpretation of evolutionary dynamics by providing an intuitive depiction of gene gain, duplication, and loss events within the broader species tree framework.

### Precise orthogroups

**Fig 8:**
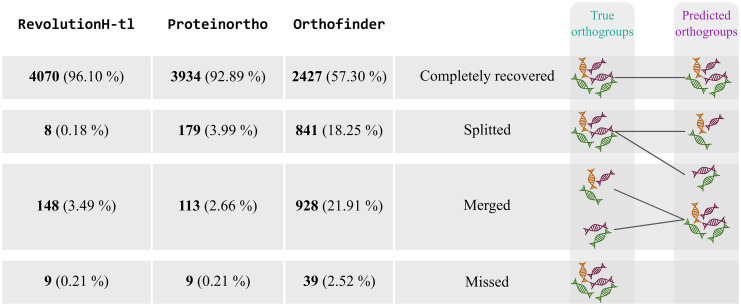
Orthogroup performance. Fully recovered orthogroups are those that exactly match a corresponding inferred orthogroup. Split orthogroups are those whose genes are distributed across multiple inferred orthogroups, indicating over-clustering. In contrast, merged orthogroups are entirely contained within a single inferred orthogroup that also includes genes from other true orthogroups, reflecting over-clustering.

Fig 8 compares the performance of REvolutionH-tl, OrthoFinder, and Proteinortho in identifying orthogroups from the dataset SaGePhy5–50. Our results show that REvolutionH-tl achieves the highest accuracy, with nearly all inferred orthogroups precisely matching their corresponding ground-truth orthogroups. In comparison, Proteinortho exhibits a moderate reduction in accuracy, recovering approximately 3% fewer orthogroups, while OrthoFinder shows a substantial performance decline, recovering nearly 40% fewer orthogroups than the ground truth.

To further investigate the sources of error, we analyze orthogroup prediction failures by quantifying two error types: orthogroups that are split across multiple predicted clusters (i.e., split orthogroups), and those that are incorrectly grouped together into a single cluster (i.e., merged orthogroups). Across all tools, merging emerges as the predominant source of error. Notably, OrthoFinder merges nearly 30% of the true orthogroups.

### High accuracy of orthology prediction

**Fig 9:**
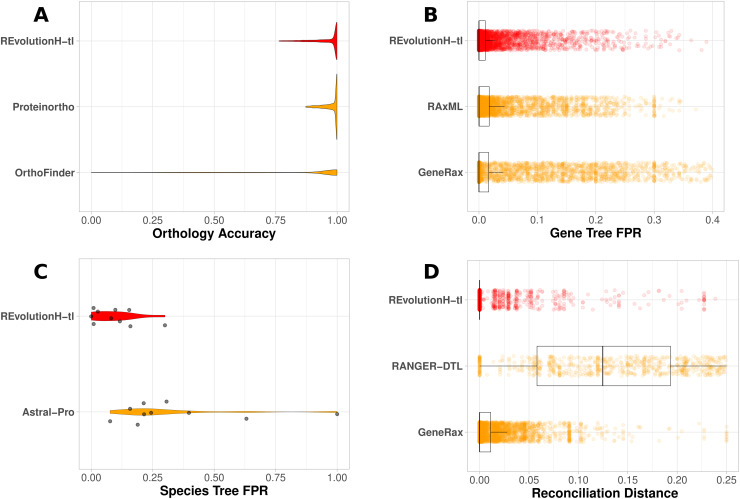
Benchmarking results for orthology prediction, gene and species tree reconstruction, and reconciliation. **(A)** Orthology accuracy. Each tool was evaluated on the same input: a collection of FASTA files from the SaGePhy5-50 dataset. All runs were performed using 16 CPUs. For OrthoFinder, the -M dendroblast parameter was used; default settings were applied for both REvolutionH-tl and Proteinortho. The average for REvolutionH-tl and Proteinortho is above 0.9 and for OrthoFinder is 0.8 **(B)** Gene tree false positive rate (FPR). FPR distributions are computed from rooted triples obtained from inferred gene trees using the SaGePhy5-50 dataset. REvolutionH-tl triples are derived from 5,736 fully recovered gene trees following reconciliation. RaxML triples are based on 5,848 inferred trees, and GeneRax triples on 5,885 trees. For RAxML and GeneRax, input consists of multiple sequence alignments (MSAs) generated with MAFFT. Both tools also require a molecular evolution model; we used the NJ-based model configuration for consistency and rooted the trees at the midpoint of the longest path. The average for REvolutionH-tl is 0.019, for RAxML is 0.027, and for GeneRax is 0.053 **(C)** Species tree false positive rate. This panel shows the FPR of rooted triples from species trees inferred from the SaGePhy5-50 dataset. Each dot represents a species tree with {5,10,...,35,40,50} taxa, inferred from up to 600 gene trees. **(D)** Reconciliation distance. Distances are calculated using the normalized PLR dissimilarity measure between the inferred evolutionary scenarios and the ground-truth histories from the SaGePhy5-50 dataset. GeneRax reconciled 5,885 gene trees, while REvolutionH-tl reconciled 5,736 gene trees, of which 3,341 are fully displayed without additional pruning. The same set of orthogroups was used as input for RANGER-DTL, which reconciles the gene trees produced by REvolutionH-tl at step 3. The same species tree was used for all tools.

Fig 9A presents the distribution of orthology prediction accuracy for OrthoFinder, Proteinortho, and REvolutionH-tl on the SaGePhy5–50 dataset. As expected, all tools exhibit high accuracy, owing to the ultrametric nature of the simulated dataset. Among them, Proteinortho achieves slightly higher accuracy than REvolutionH-tl in recovering orthologous gene pairs, while OrthoFinder demonstrates a significantly lower accuracy in orthology inference.

This performance trend remains consistent when results are stratified by the simulation parameters used to generate the evolutionary scenarios, particularly the rates of gene duplication and loss. For a more comprehensive analysis of orthology prediction under varying evolutionary conditions, we refer the reader to [27, Fig 5].

**Fig 10:**
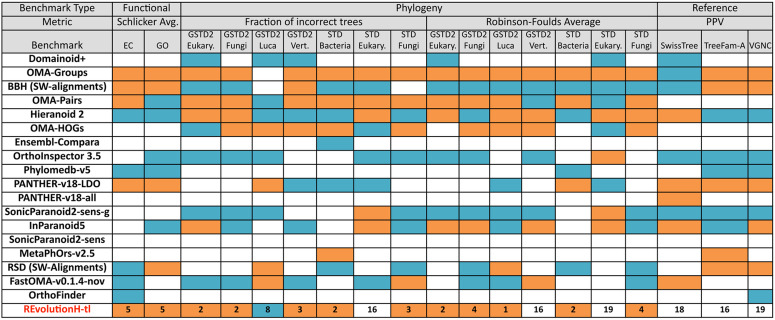
Precision of orthology prediction. The average precision of REvolutionH-tl and 18 tools from the QfO benchmark dataset 2022_02 is in orange for the top 25% tools and in blue for the top 50% tools. Benchmark types are phylogeny-based, function-based, or reference orthology-based. Rankings for REvolutionH-tl are included. Rankings for the other tools are in S2 Appendix.

Additionally, REvolutionH-tl is the second most precise tool assessing orthology prediction using the Quest for Orthologs (QfO) web service [61]. Fig 10 shows that the precision of REvolutionH-tl predictions lies 12 times in the top 25% tools, and once in the top 50%, for a total of 13 tests and 19 tools. Among the other tools, only OMA-Groups outperforms REvolutionH-tl, lying 17 times in the top 25% of tools. However, REvolutionH-tl underperforms in the reference gene tree tests. Because these benchmarks evaluate orthology predictions against orthology relations derived from reference gene trees in public databases, discrepancies do not necessarily imply low precision of REvolutionH-tl. Reference gene trees are themselves inferred evolutionary hypotheses and may contain reconstruction errors that propagate to the derived orthology relations. Consequently, differences between REvolutionH-tl and these benchmarks may also reflect alternative phylogenetic reconstructions rather than absolute inference failures.

As described in the methodology, we focus our analysis of the QfO benchmark on precision metrics, since recall estimates are biased toward tools that predict a larger number of orthology relations, regardless of their biological validity. For completeness, the recall results are provided in S2 Appendix

### Accuracy for gene and species tree reconstruction

Fig 9B compares the distribution of false positive rates (FPR) for gene tree triples produced by three methods: REvolutionH-tl, RaxML, and GeneRax. All three tools maintain a low FPR, indicating reliable performance in capturing true topological signals. Notably, both RaxML and GeneRax rely on maximum likelihood methods and use the LG substitution model - the same model applied to simulate sequences in the SaGePhy5–50 data set. Based on this methodological alignment, one might expect these tools to outperform REvolutionH-tl. However, it is noteworthy that REvolutionH-tl also achieves a comparably low FPR, despite relying on a simpler and faster approach.

Fig 9C shows the FPR distributions for species tree triples inferred by REvolutionH-tl and ASTRAL-Pro on the SaGePhy5–50 dataset. For each number of species in the set {5,10,15,...,35,40,50}, approximately 600 gene trees are used to infer a single species tree. In this comparison, REvolutionH-tl operates on partially resolved gene trees, whereas ASTRAL-Pro introduces random binary resolutions for all polytomies. This difference in treatment of unresolved nodes likely contributes to the superior accuracy of species trees inferred by REvolutionH-tl, as it avoids introducing potentially misleading topological artifacts through arbitrary resolutions.

Additionally, we measured tree distance using the Robinson–Foulds (RF) metric [64] for the same datasets as above (see S3 Appendix). Under this metric, REvolutionH-tl exhibits greater discordance with the true gene trees, indicating larger differences in terms of bipartitions (splits). In contrast, the rooted triplet analysis shows a low false positive rate, indicating that most local ancestor–descendant relationships are correctly recovered. This discrepancy reflects the fact that RF distance captures global topological differences, whereas triplet-based metrics evaluate local phylogenetic relationships, suggesting that revolutionhtl preserves local evolutionary structure while differing more at the level of overall tree topology.

### Precise tree reconciliation

Fig 9D presents the distribution of reconciliation distances for REvolutionH-tl, RANGER-DTL, and GeneRax, evaluated on the SaGePhy5–50 dataset. All three tools demonstrate the ability to reconcile gene trees with the true species tree to a reasonable extent. However, the distribution for REvolutionH-tl is notably concentrated near zero, indicating that the evolutionary scenarios it produces are, on average, closer to the ground-truth evolutionary history. In contrast, the reconciliations generated by GeneRax and RANGER-DTL exhibit higher variability and larger distances, suggesting greater divergence from the simulated evolutionary scenarios.

### Fast reconstruction of evolution

**Fig 11:**
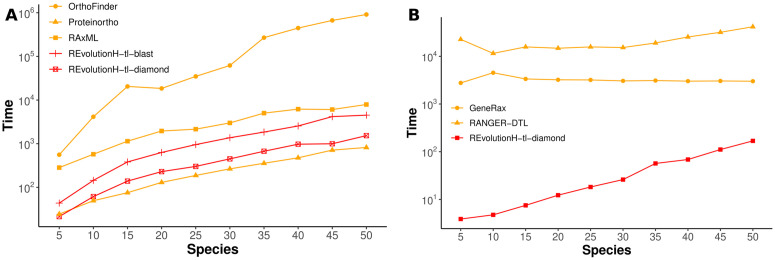
Running times for trees reconstruction and reconciliation on synthetic dataset. These figures show the total execution time needed for the analysis of the SaGePhy5-50 dataset, measured in seconds. **(A)** Time performance for gene tree reconstruction and orthology inference. All the tools for this analysis were executed using 16 cpus. For REvolutionH-tl we only consider running times of steps one to three. **(B)** Time performance for three reconciliation. We consider only step 6 of REvolutionH-tl workflow.

The runtime performance of the tools for gene tree reconstruction included in our benchmarking analysis is shown in Fig 11A. REvolutionH-tl successfully completes the full analysis of genomes from the SaGePhy5–50 dataset. Notably, even for the largest dataset, REvolutionH-tl completes the analysis in under one hour. This represents a substantial improvement over the previous release of the tool, where the same analysis required several hours to complete [27].

In comparison with other tools, REvolutionH-tl demonstrates superior runtime performance relative to both RAxML and OrthoFinder. As expected, Proteinortho completes its task more quickly than REvolutionH-tl; however, this speed comes at the cost of limited functionality, as Proteinortho only predicts orthology relations and does not infer gene or species phylogenies, nor perform reconciliations. Fig 11B presents the runtimes specifically for the reconciliation step. In this comparison, REvolutionH-tl clearly outperforms both RANGER-DTL and GeneRax, highlighting its efficiency in producing evolutionary scenarios while maintaining high accuracy.

**Fig 12:**
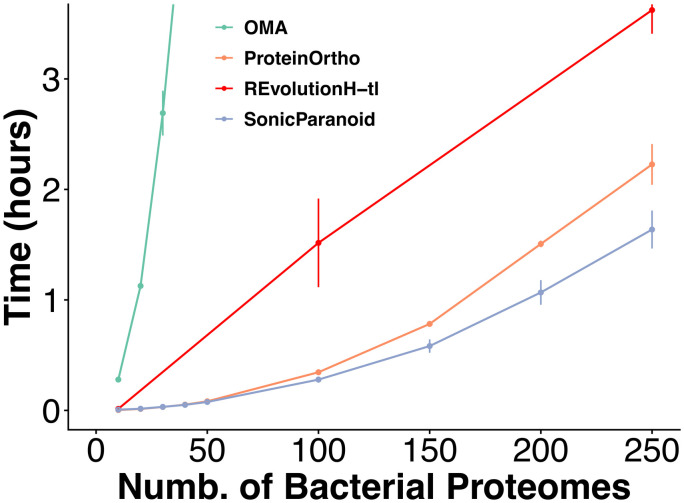
Running times for orthology inference on bacterial genomes. This figure shows the total execution time required for the analysis of the prt10-250 dataset, measured in hours.

Fig 12 shows runtime performance for large-scale orthology prediction in real bacterial genomes. In this test, REvolutionH-tl requires more time than Proteinortho and SonicParanoid. However, as with the synthetic dataset, REvolutionH-tl provides not only orthology predictions but also gene trees, enabling validation of the inferred evolutionary relationships.

## Discussion

REvolutionH-tl introduces a novel, efficient, and comprehensive framework for reconstructing evolutionary histories from sequence data. Unlike traditional methods that require precomputed gene or species trees and multiple specialized tools, REvolutionH-tl delivers an end-to-end platform that integrates orthology prediction, phylogenetic inference, and reconciliation into a unified system. Its core relies on the theoretical foundation of best match graphs (BMGs), allowing robust orthogroup and orthology detection through graph-based heuristics, which are both scalable and accurate.

Across benchmarking analyses, REvolutionH-tl consistently demonstrates high performance in reconstructing orthogroups and inferring orthology relationships. It matches or exceeds the accuracy of widely used tools such as OrthoFinder and Proteinortho while maintaining a significantly reduced computational footprint. Particularly in large simulated datasets with known ground truth, REvolutionH-tl recovers nearly all true orthogroups with minimal merging or splitting errors—an essential quality for downstream evolutionary analyses. The use of BMGs ensures that orthology is inferred based on evolutionary proximity rather than mere sequence similarity, mitigating common pitfalls in reciprocal best-hit approaches.

A central innovation of REvolutionH-tl lies in its inference of event-labeled gene trees and a *de novo* species tree, guided by orthology assignments rather than alignment likelihoods. Despite not using maximum likelihood or Bayesian methods, REvolutionH-tl produces gene trees with comparably low false-positive rates, as observed in evaluations against tools like RAxML and GeneRax. Furthermore, the tool’s reconciliation procedure consistently maps gene trees to species trees with high accuracy. Notably, reconciliation distances between inferred and true scenarios are lowest for REvolutionH-tl, affirming the biological plausibility of the reconstructed histories.

Beyond accuracy and efficiency, REvolutionH-tl sets itself apart as the first platform to provide built-in, publication-quality visualizations that facilitate the interpretation of evolutionary histories. Through intuitive plots, users can explore genome complexity, orthogroup statistics, and gene tree reconciliations in both global and orthogroup-specific contexts. These visualizations enhance the interpretability of results by embedding gene trees within species tree frameworks, clearly annotating events such as gene duplications, speciations, and losses. This level of graphical integration is unmatched by other tools, which often require manual scripting or external software to generate comparable insights.

The tool’s visual capabilities serve not only as a means of validation but also as a powerful communication aid, making it easier for biologists to trace evolutionary trajectories and gene family dynamics. With commands like plot_summary and plot_reconciliation, users can quickly generate diagrams that summarize gene content evolution, orthology types, and lineage-specific innovations—key features for comparative genomics and evolutionary studies.

In summary, REvolutionH-tl 2.0 advances the field by delivering an integrated, graph-driven, and visually rich platform for evolutionary inference. Its balance of theoretical rigor, practical speed, and interpretability makes it a valuable resource for researchers exploring gene family evolution at scale. Future work will expand support for modeling horizontal gene transfer and enhance sensitivity to highly divergent sequences, further cementing REvolutionH-tl’s role as a cornerstone in computational evolutionary biology.

## Supporting information

S1 Appendix Theoretical advantage of best matches over best hits. This panel illustrates the superiority of best match graphs for identifying evolutionary relationships and enabling early detection of errors in orthology and best hit inference by graph-based methods. Each node represents a gene, and the color indicates the species in which it is found. (A) shows a best match graph whose symmetric edges form a path on four nodes. Assuming these reciprocal relations correspond to orthology leads to a well-known contradiction: orthology relations cannot form a path on four genes [51]. Furthermore, the informative triples described in Section *Best match graphs and gene trees* allow reconstruction of the gene tree shown in (B), demonstrating that genes *x* and *v* are paralogs rather than orthologs. When inferring bidirectional best hits from sequence similarity data, it is possible to obtain a graph across three species such as the one shown in (C). This graph not only fails to represent valid orthology (since it forms a path on four nodes), but also cannot be explained by any gene tree. More generally, data-driven best hit inference may produce graphs like (C), whose informative triples reveal inconsistencies with any gene tree, indicating that the graph must be edited to restore phylogenetic consistency. Examples (A–C) were adapted from [47].
(PDF)

S2 Appendix QfO performance results. We include several figures in this file: (*Ranking of precision of orthology prediction.*) The average precision of REvolutionH-tl and 18 tools from the QfO benchmark dataset 2022_02 is in orange for the top 25% tools and in blue for the top 50% tools. Rankings for all tools and all benchmarks are included. (*STD-Bacteria*) x-axis detects the recall measured as completed tree samples; y-axis detects the average Robinson-Foulds distance. (*STD-Fungi*) x-axis detects the recall measured as completed tree samples; y-axis detects the average Robinson-Foulds distance. (*STD-Eukaryota*) x-axis detects the recall measured as completed tree samples; y-axis detects the average Robinson-Foulds distance. (*GSTD2-Eukaryota*) x-axis detects the recall measured as completed tree samples; y-axis detects the average Robinson-Foulds distance. (*GSTD2-Fungi*) x-axis detects the recall measured as completed tree samples; y-axis detects the average Robinson-Foulds distance. (*GSTD2-Luca*) x-axis detects the recall measured as completed tree samples; y-axis detects the average Robinson-Foulds distance. (*GSTD2-Vertebrata*) x-axis detects the recall measured as completed tree samples; y-axis detects the average Robinson-Foulds distance. (*VGNC*) x-axis detects True Positive Rate (TPR); y-axis detects precision measured as Positive Predictive Value (PPV). (*TreeFam-A*) x-axis detects True Positive Rate (TPR); y-axis detects precision measured as Positive Predictive Value (PPV). (*SwissTree*) x-axis detects True Positive Rate (TPR); y-axis detects precision measured as Positive Predictive Value (PPV). (*GOtest*) x-axis detects True Positive Rate (TPR); y-axis detects precision measured as Positive Predictive Value (PPV). (*ECtest*) x-axis detects recall measured as number of ortholog relations; y-axis detects precision measured as average Schlicker Similarity.
(PDF)

S3 AppendixDiscordance between inferred and true phylogenies.Robinson-Foulds distance is compued for the rooted trees inferred using different methodologies for gene (left) and species trees (right) on the SaGePhy5–50 dataset. REvolutionH-tl gene trees correspond to the 5,736 fully recovered gene trees following reconciliation. RaxML shows 5,848 inferred trees, and GeneRax reconstructs 5,885 trees. For RAxML and GeneRax, input consists of multiple sequence alignments (MSAs) generated with MAFFT. Both tools also require a molecular evolution model; we used the NJ-based model configuration for consistency and rooted the trees at the midpoint of the longest path. The average for REvolutionH-tl is 0.144, for RAxML is 0.037, and for GeneRax is 0.144. On the right side, each dot represents a species tree with {5,10,...,35,40,50} taxa, inferred from up to 600 gene trees produced at the step 3 of REvolutionH-tl.(PDF)
