# Supplementary material for: REvolutionH-tl 2.0: A fast and robust tool for decoding evolutionary gene histories
Source: PLoS Comput Biol. 2026 Aug 12;22(8):e1013017. doi: 10.1371/journal.pcbi.1013017 (PMC13485135; doi:10.1371/journal.pcbi.1013017)
Supplement: Supplementary file 1 [file pcbi.1013017.s001.pdf]

S1 Appendix. Theoretical advantage of best matches over best hits

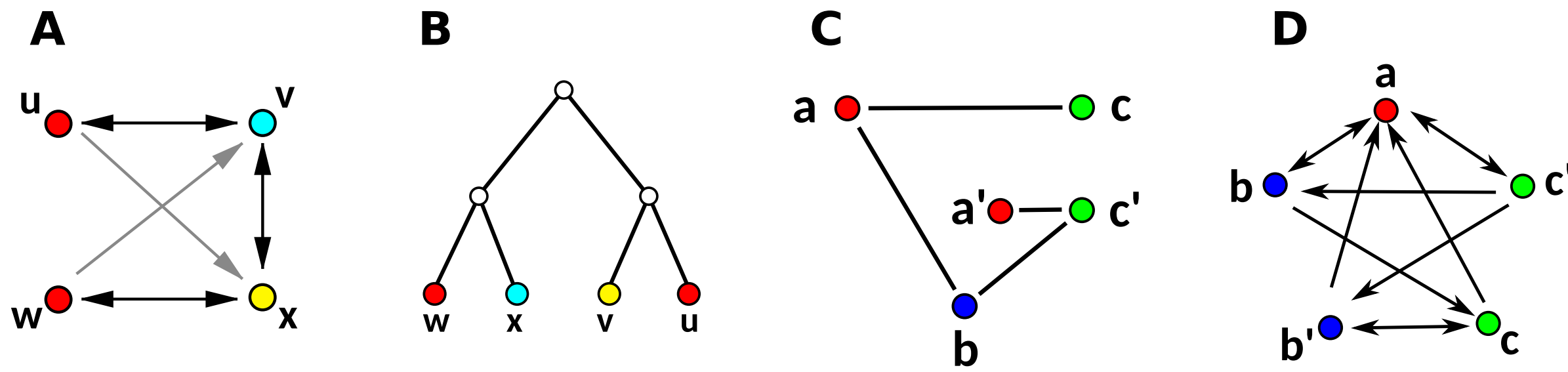

S1-Fig 1. Theoretical advantage of best matches over best hits. This panel illustrates the superiority of best match graphs for identifying evolutionary relationships and enabling early detection of errors in orthology and best hit inference by graph-based methods. Each node represents a gene, and the color indicates the species in which it is found. (A) shows a best match graph whose symmetric edges form a path on four nodes. Assuming these reciprocal relations correspond to orthology leads to a well-known contradiction: orthology relations cannot form a path on four genes [1]. Furthermore, the informative triples described in Section *Best match graphs and gene 1067 trees* allow reconstruction of the gene tree shown in (B), demonstrating that genes x and v are paralogs rather than orthologs. When inferring bidirectional best hits from sequence similarity data, it is possible to obtain a graph across three species such as the one shown in (C). This graph not only fails to represent valid orthology (since it forms a path on four nodes), but also cannot be explained by any gene tree. More generally, data-driven best hit inference may produce graphs like (C), whose informative triples reveal inconsistencies with any gene tree, indicating that the graph must be edited to restore phylogenetic consistency. Examples (A–C) were adapted from [2].

References

[1] Hellmuth M, Hernandez-Rosales M, Huber KT, Moulton V, Stadler PF, Wieseke N. Orthology relations, symbolic ultrametrics, and cographs. Journal of Mathematical Biology. 2013;66(1):399–420. doi:10.1007/s00285-012-0525-x.

[2] Geiß M, Chávez E, González Laffitte M, López Sánchez A, Stadler BMR, Valdivia DI, et al. Best match graphs. Journal of Mathematical Biology. 2019;78(7):2015–2057. doi:10.1007/s00285-019-01332-9.
