# Supplementary material for: REvolutionH-tl 2.0: A fast and robust tool for decoding evolutionary gene histories
Source: PLoS Comput Biol. 2026 Aug 12;22(8):e1013017. doi: 10.1371/journal.pcbi.1013017 (PMC13485135; doi:10.1371/journal.pcbi.1013017)
Supplement: Supplementary file 2 [file pcbi.1013017.s002.pdf]

S2 Appendix. QfO performance results

QfO precision ranking

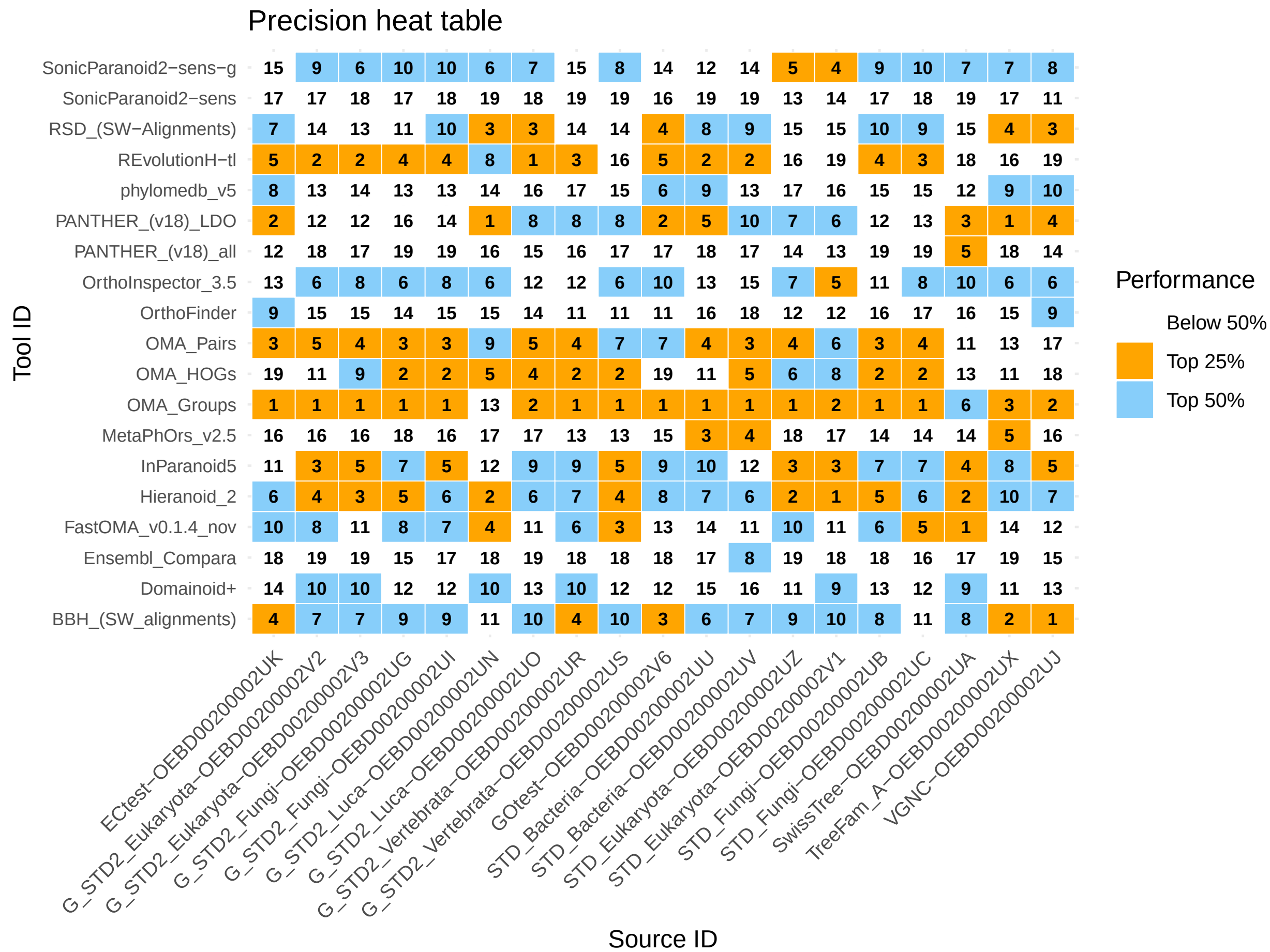

Tool ID	ECtest–OEBD00200002UK	G_STD2_Eukaryota–OEBD00200002V2	G_STD2_Eukaryota–OEBD00200002V3	G_STD2_Fungi–OEBD00200002UG	G_STD2_Fungi–OEBD00200002UI	G_STD2_Luca–OEBD00200002UN	G_STD2_Luca–OEBD00200002UO	G_STD2_Vertebrata–OEBD00200002UR	G_STD2_Vertebrata–OEBD00200002US	GOtest–OEBD00200002V6	STD_Bacteria–OEBD00200002UU	STD_Bacteria–OEBD00200002UV	STD_Eukaryota–OEBD00200002UZ	STD_Eukaryota–OEBD00200002V1	STD_Fungi–OEBD00200002UB	STD_Fungi–OEBD00200002UC	SwissTree–OEBD00200002UA	TreeFam_A–OEBD00200002UX	VGNC–OEBD00200002UJ
SonicParanoid2–sens–g	15	9	6	10	10	6	7	15	8	14	12	14	5	4	9	10	7	7	8
SonicParanoid2–sens	17	17	18	17	18	19	18	19	19	16	19	19	13	14	17	18	19	17	11
RSD_(SW–Alignments)	7	14	13	11	10	3	3	14	14	4	8	9	15	15	10	9	15	4	3
REvolutionH–tl	5	2	2	4	4	8	1	3	16	5	2	2	16	19	4	3	18	16	19
phylomedb_v5	8	13	14	13	13	14	16	17	15	6	9	13	17	16	15	15	12	9	10
PANTHER_(v18)_LDO	2	12	12	16	14	1	8	8	8	2	5	10	7	6	12	13	3	1	4
PANTHER_(v18)_all	12	18	17	19	19	16	15	16	17	17	18	17	14	13	19	19	5	18	14
OrthoInspector_3.5	13	6	8	6	8	6	12	12	6	10	13	15	7	5	11	8	10	6	6
OrthoFinder	9	15	15	14	15	15	14	11	11	11	16	18	12	12	16	17	16	15	9
OMA_Pairs	3	5	4	3	3	9	5	4	7	7	4	3	4	6	3	4	11	13	17
OMA_HOGs	19	11	9	2	2	5	4	2	2	19	11	5	6	8	2	2	13	11	18
OMA_Groups	1	1	1	1	1	13	2	1	1	1	1	1	1	2	1	1	6	3	2
MetaPhOrs_v2.5	16	16	16	18	16	17	17	13	13	15	3	4	18	17	14	14	14	5	16
InParanoid5	11	3	5	7	5	12	9	9	5	9	10	12	3	3	7	7	4	8	5
Hieranoid_2	6	4	3	5	6	2	6	7	4	8	7	6	2	1	5	6	2	10	7
FastOMA_v0.1.4_nov	10	8	11	8	7	4	11	6	3	13	14	11	10	11	6	5	1	14	12
Ensembl_Compara	18	19	19	15	17	18	19	18	18	18	17	8	19	18	18	16	17	19	15
Domainoid+	14	10	10	12	12	10	13	10	12	12	15	16	11	9	13	12	9	11	13
BBH_(SW_alignments)	4	7	7	9	9	11	10	4	10	3	6	7	9	10	8	11	8	2	1

S2-Fig 1. Ranking of precision of orthology prediction. The average precision of REvolutionH-tl and 18 tools from the QfO benchmark dataset 2022_02 is in orange for the top 25% tools and in blue for the top 50% tools. Rankings for all tools and all benchmarks are included.

Phylogeny-based definition benchmarks

Species Tree Discordance Benchmark

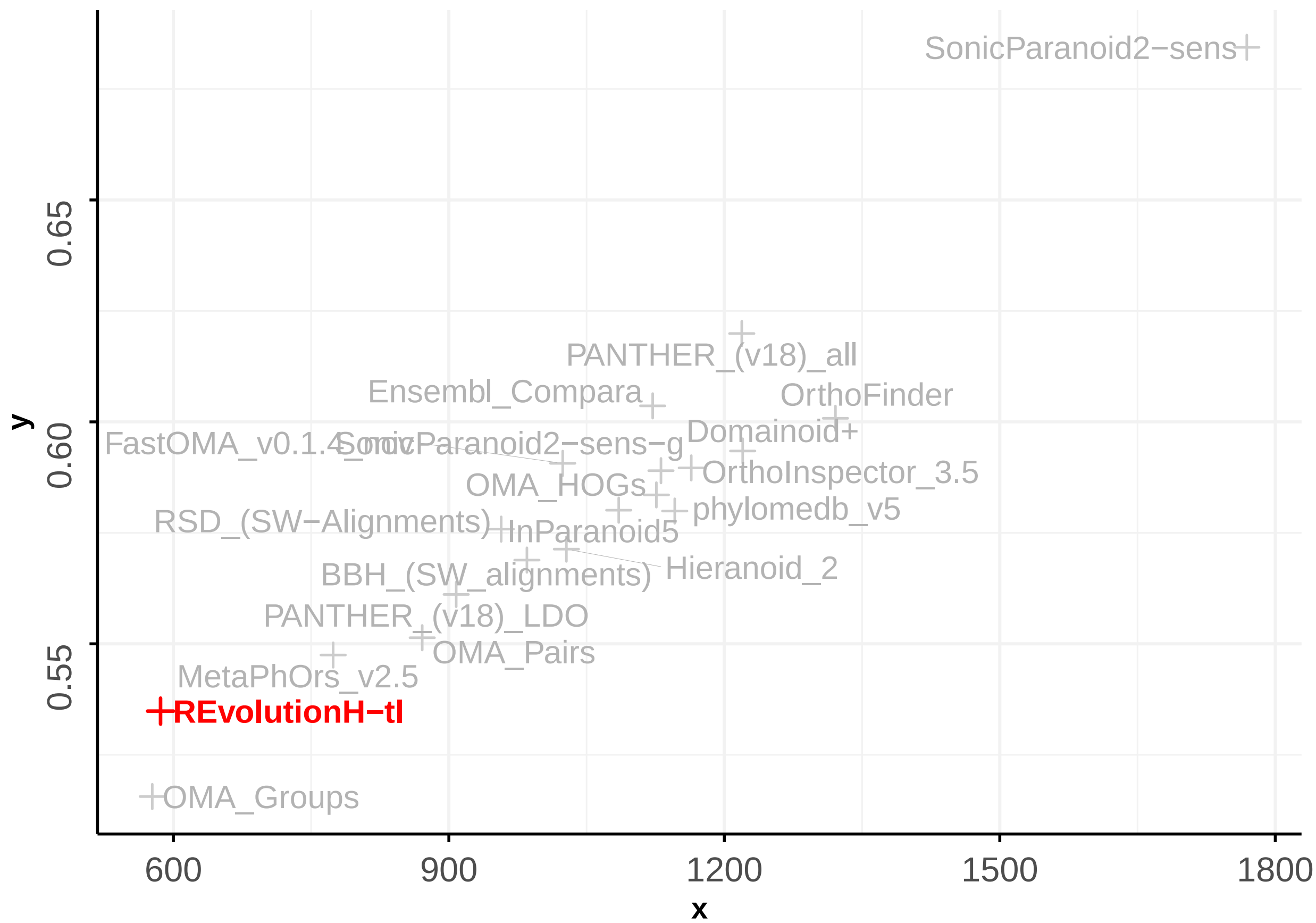

S2-Fig 2. STD-Bacteria x-axis detects the recall measured as completed tree samples; y-axis detects the average Robinson-Foulds distance.

Generalized Species Tree Discordance Benchmark

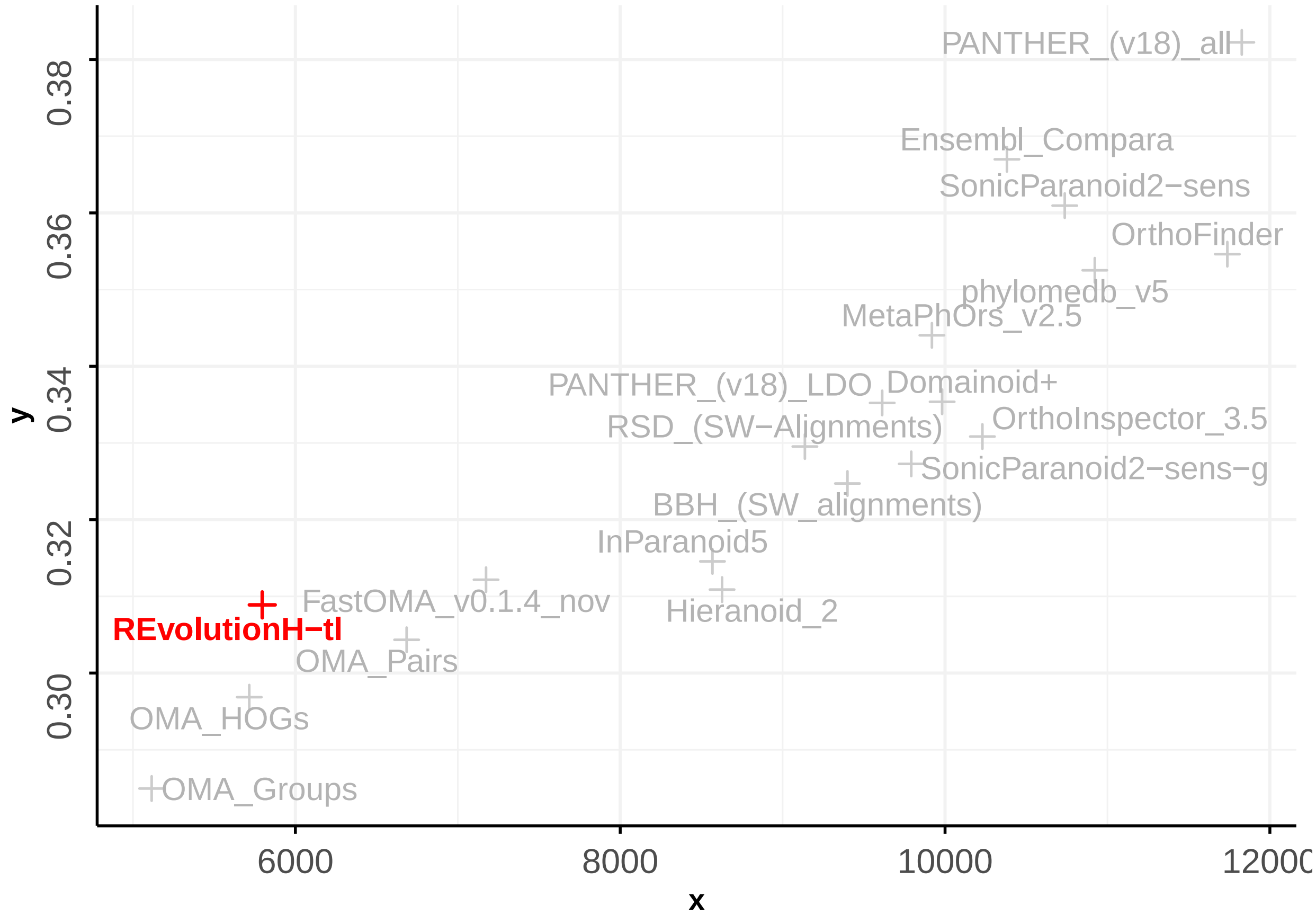

S2-Fig 3. STD-Fungi x-axis detects the recall measured as completed tree samples; y-axis detects the average Robinson-Foulds distance.

Reference orthology-based benchmarks

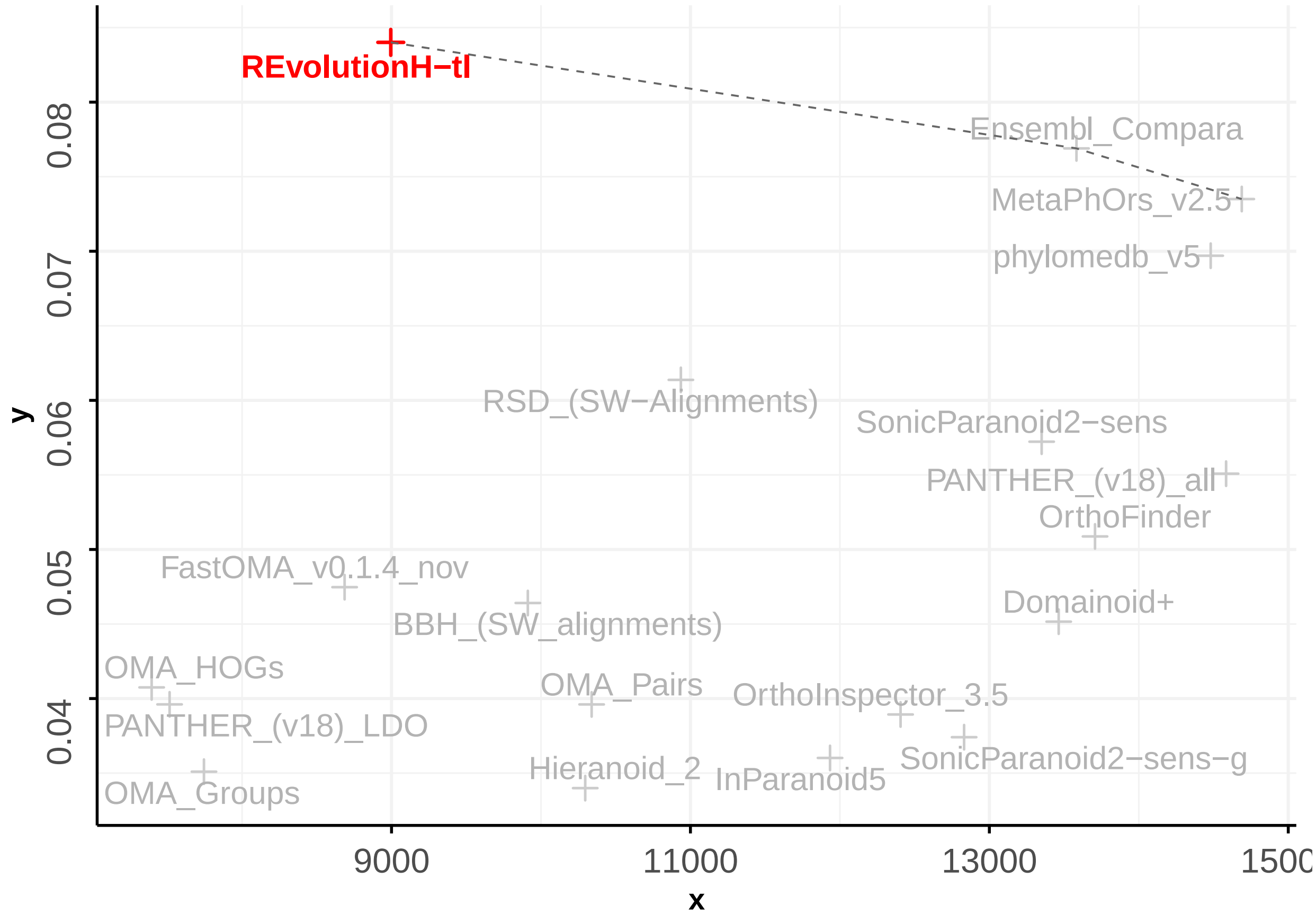

S2-Fig 4. STD-Eukaryota x-axis detects the recall measured as completed tree samples; y-axis detects the average Robinson-Foulds distance.

Function-based benchmarks

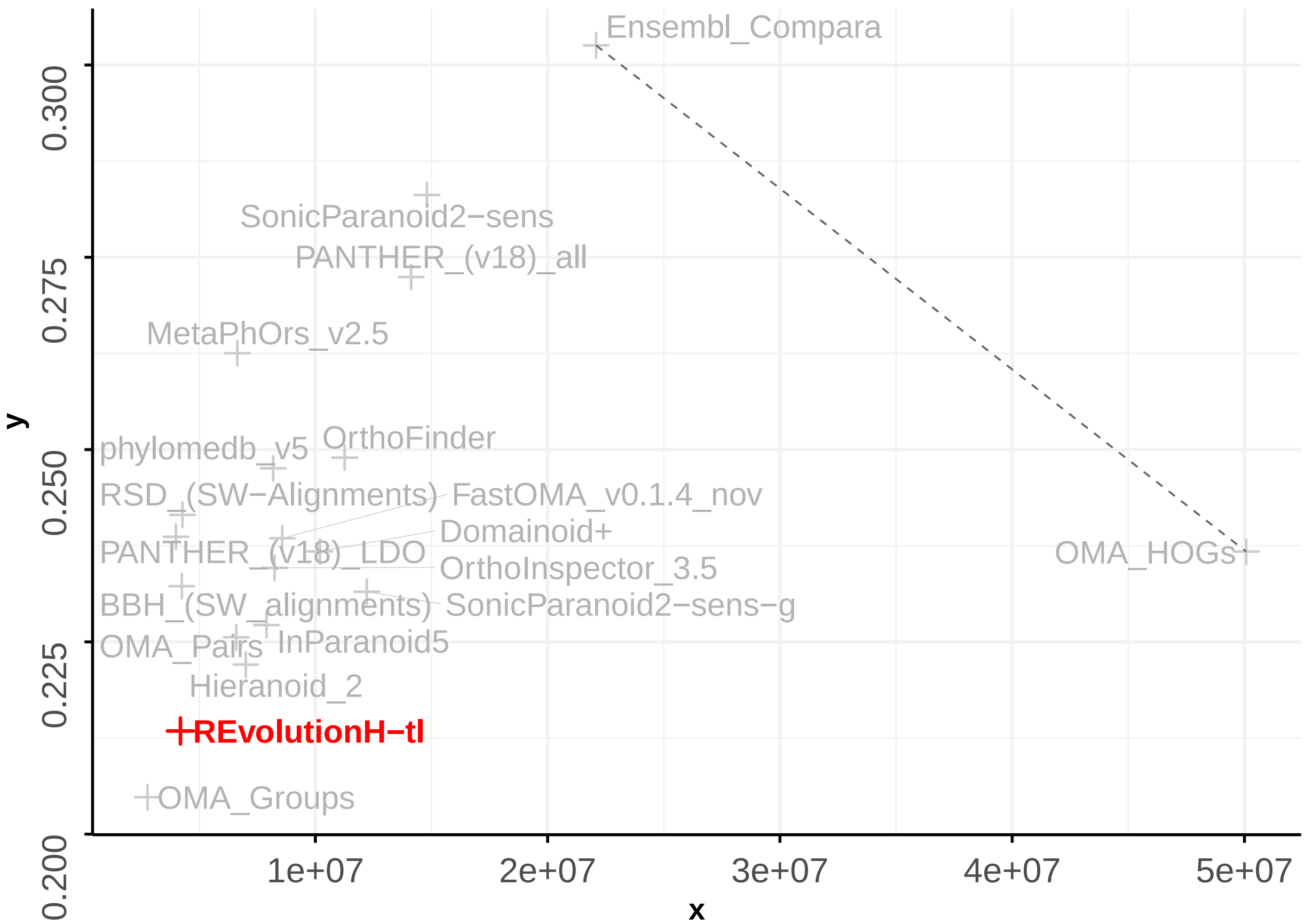

S2-Fig 5. GSTD2-Eukaryota x-axis detects the recall measured as completed tree samples; y-axis detects the average Robinson-Foulds distance.

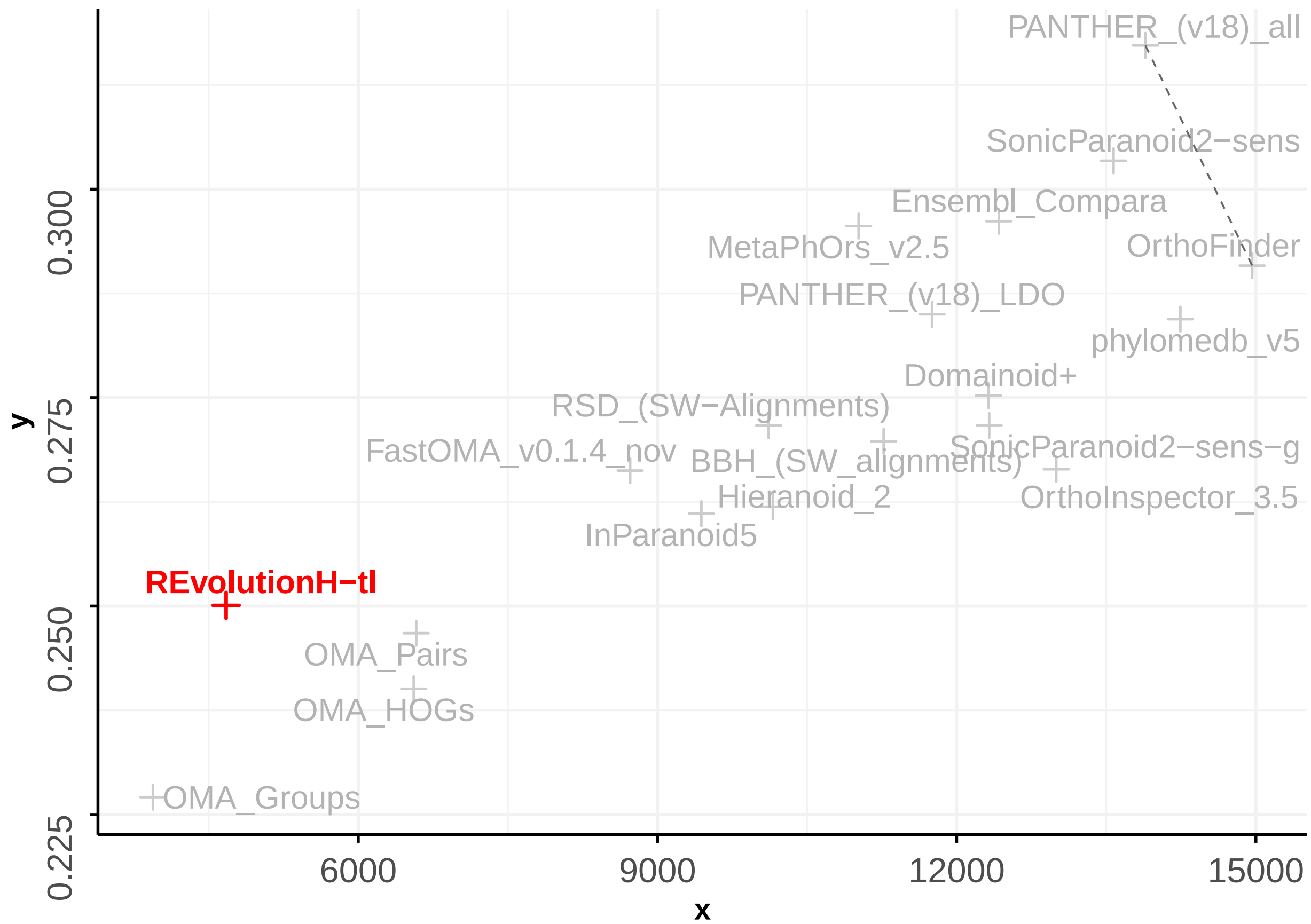

S2-Fig 6. GSTD2-Fungi x-axis detects the recall measured as completed tree samples; y-axis detects the average Robinson-Foulds distance.

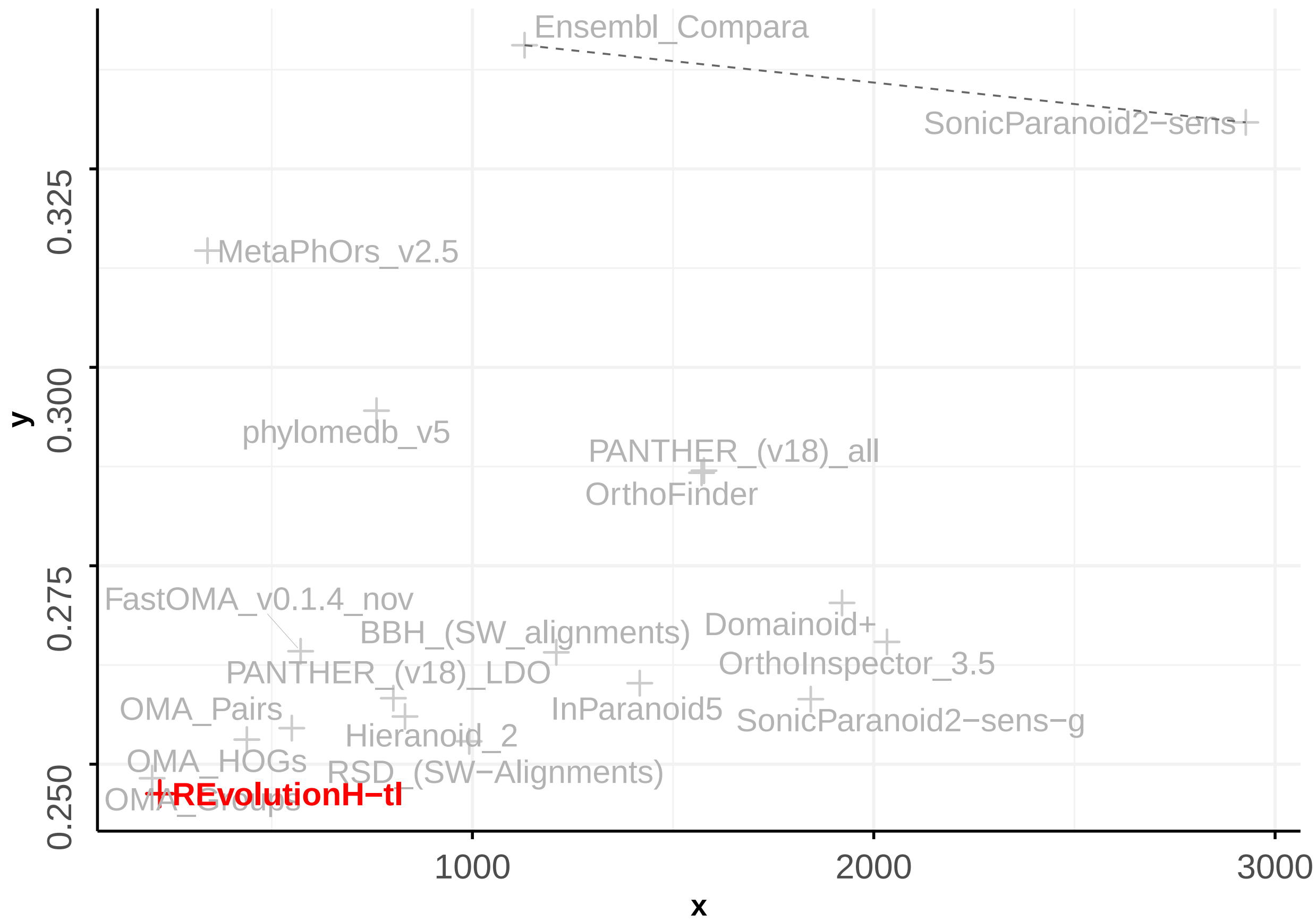

S2-Fig 7. GSTD2-Luca x-axis detects the recall measured as completed tree samples; y-axis detects the average Robinson-Foulds distance.

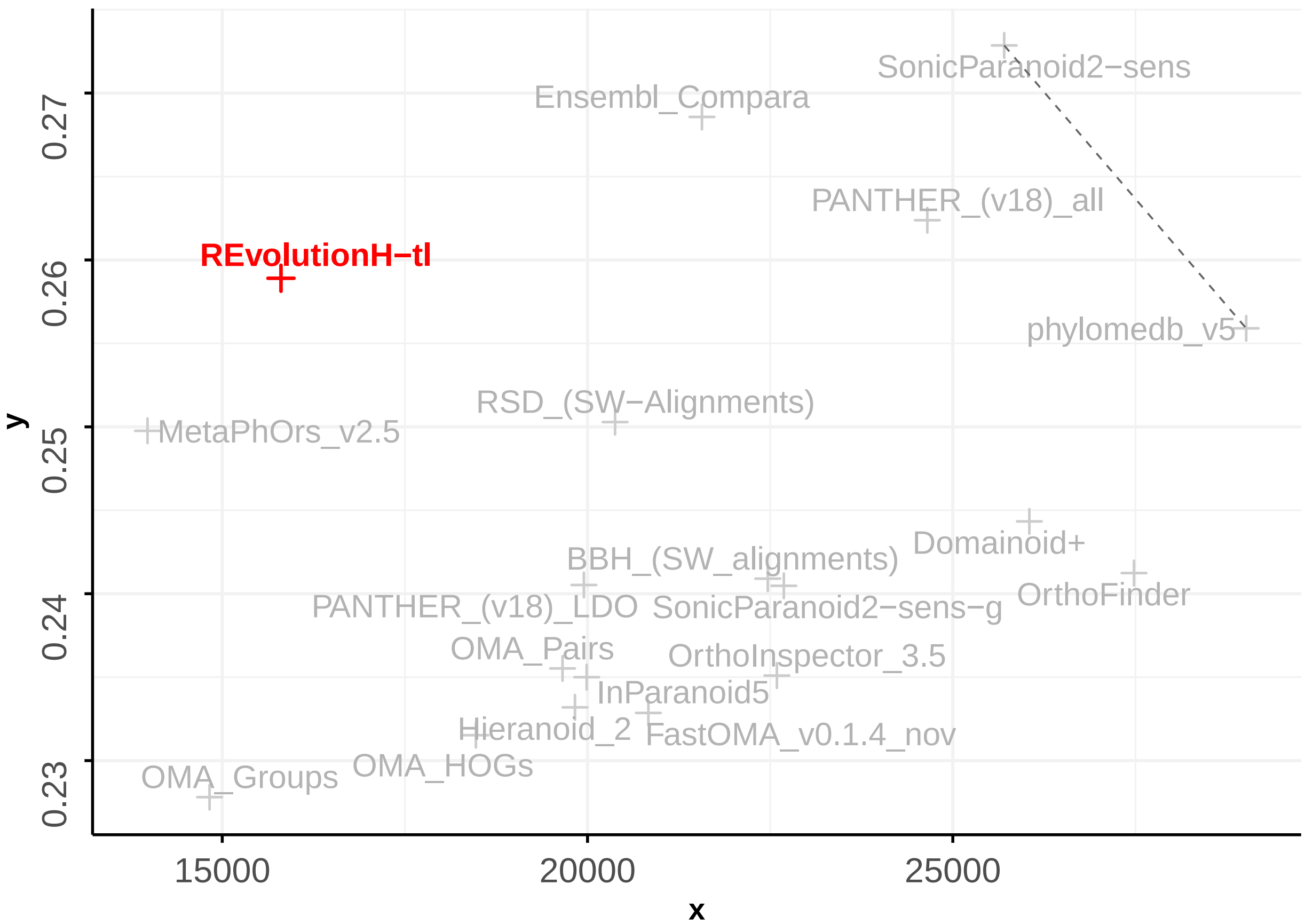

S2-Fig 8. GSTD2-Vertebrata x-axis detects the recall measured as completed tree samples; y-axis detects the average Robinson-Foulds distance.

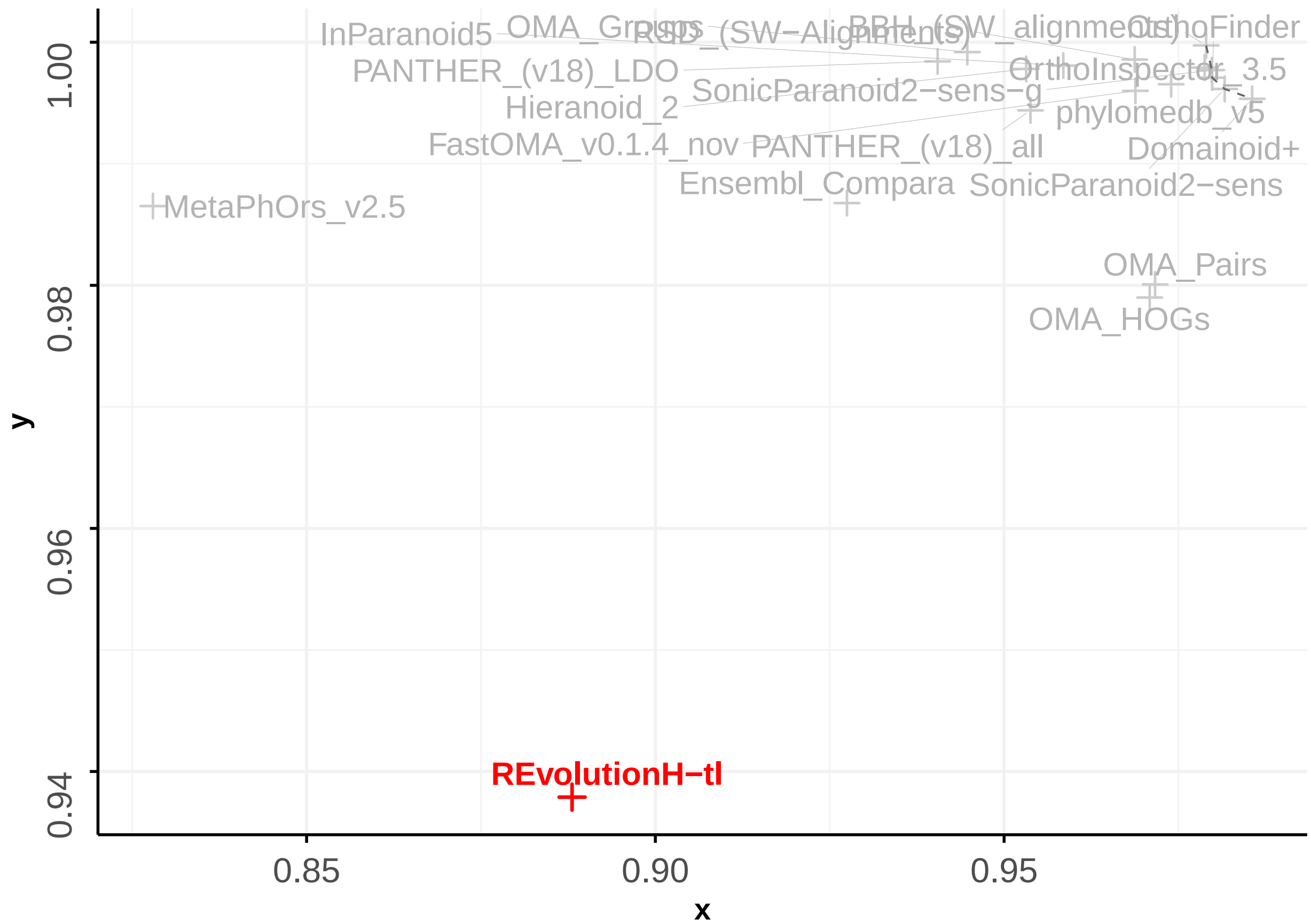

S2-Fig 9. VGNC x-axis detects True Positive Rate (TPR); y-axis detects precision measured as Positive Predictive Value (PPV).

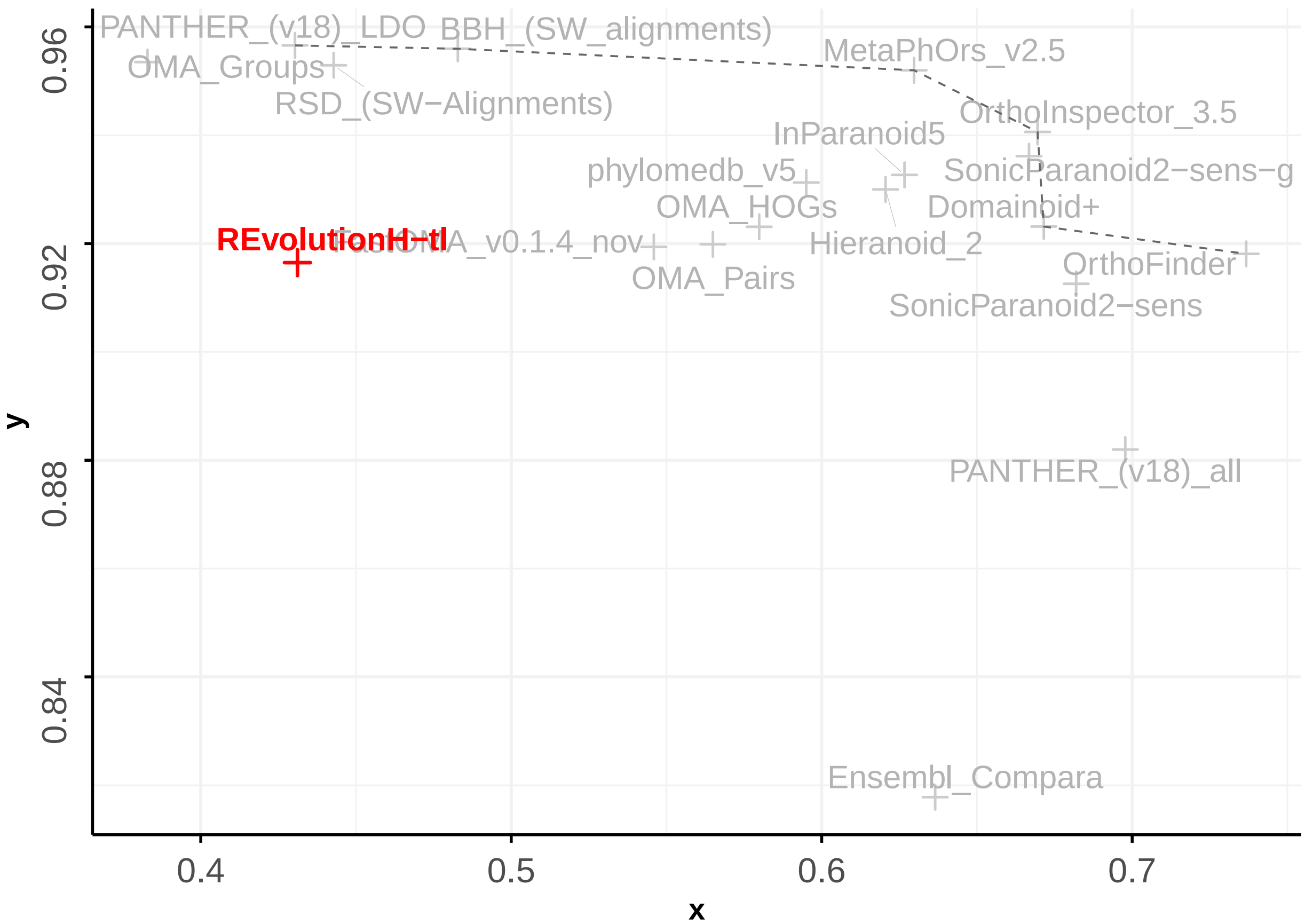

S2-Fig 10. TreeFam-A x-axis detects True Positive Rate (TPR); y-axis detects precision measured as Positive Predictive Value (PPV).

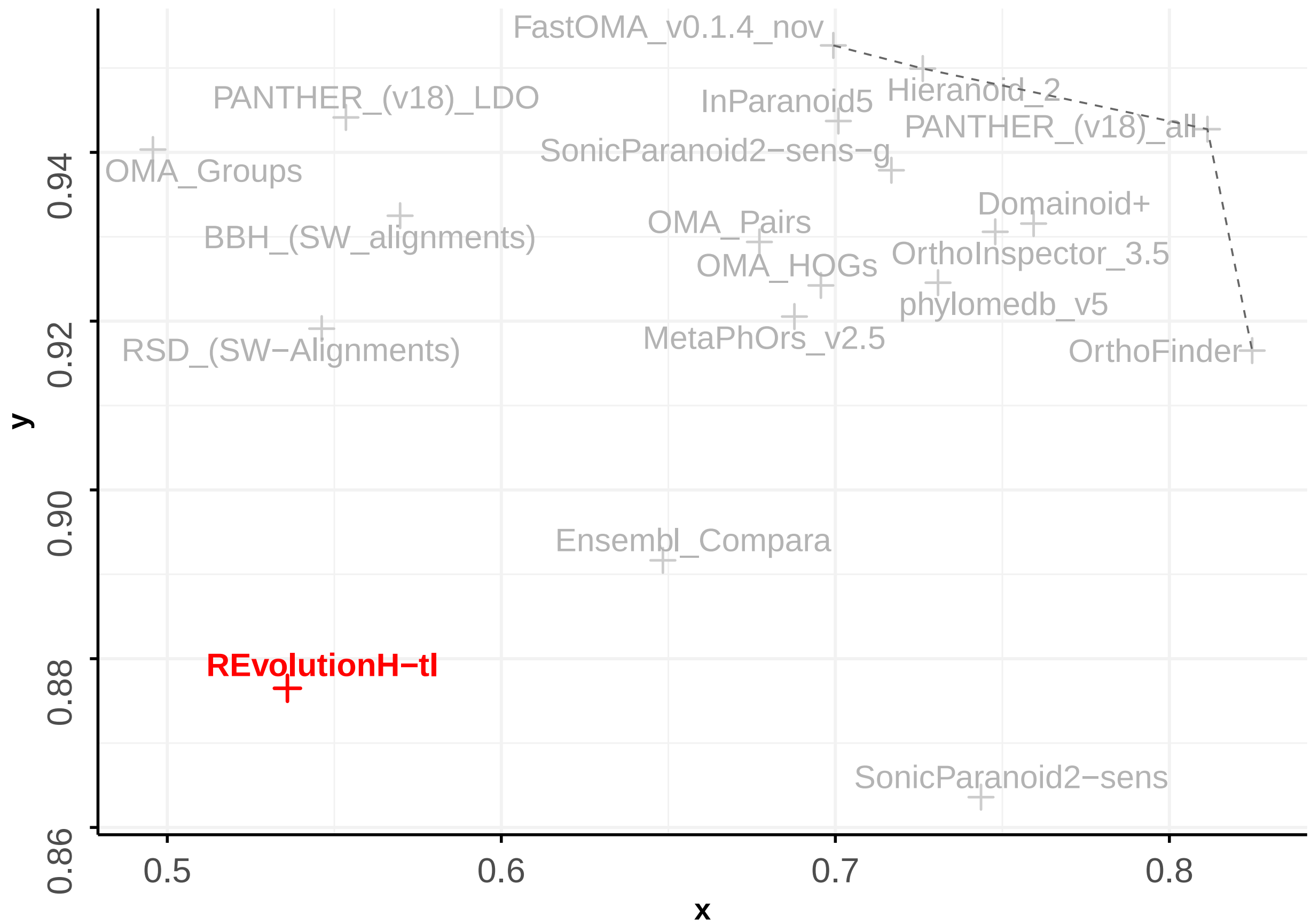

S2-Fig 11. SwissTree x-axis detects True Positive Rate (TPR); y-axis detects precision measured as Positive Predictive Value (PPV).

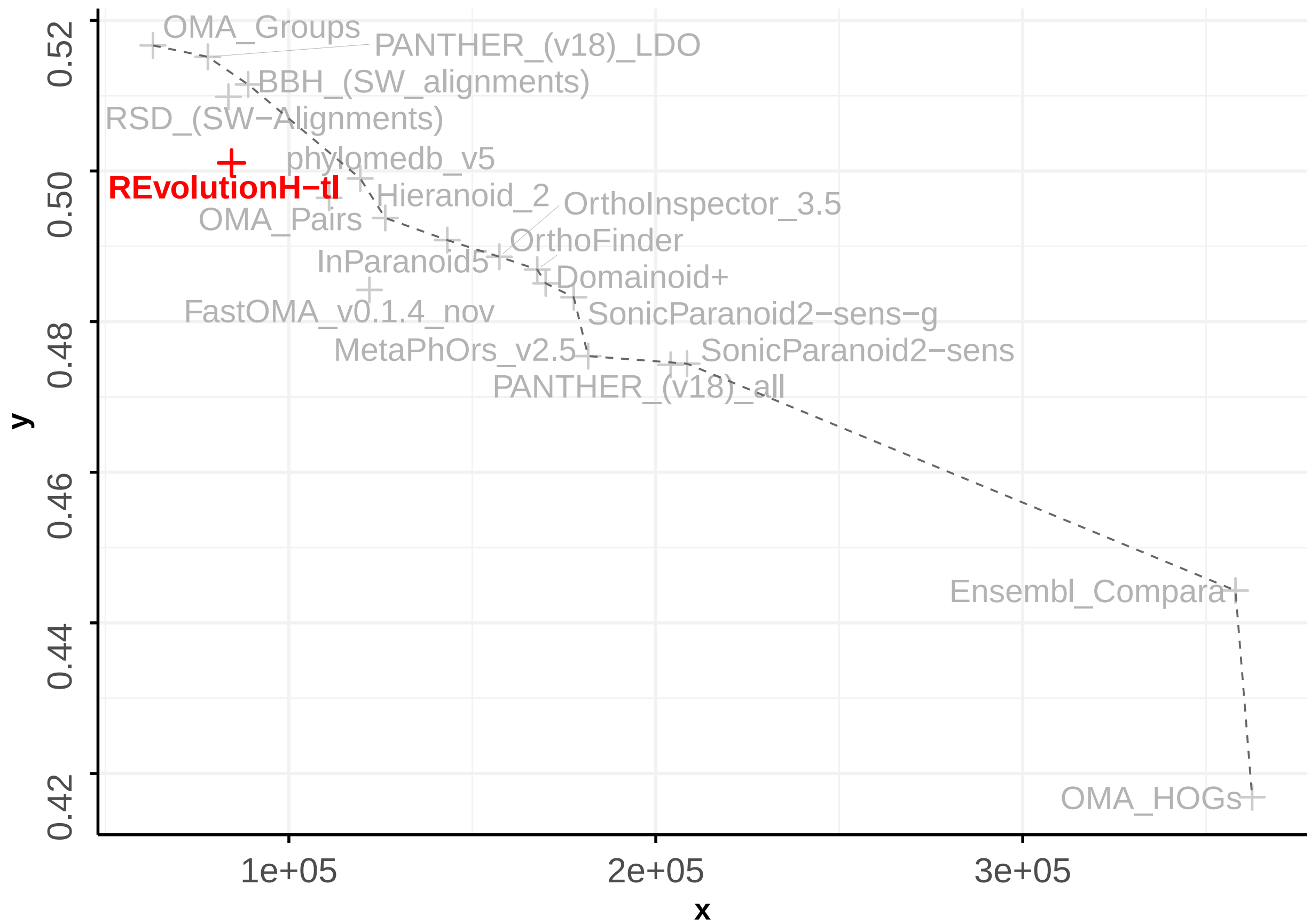

S2-Fig 12. GOtest x-axis detects True Positive Rate (TPR); y-axis detects precision measured as Positive Predictive Value (PPV).

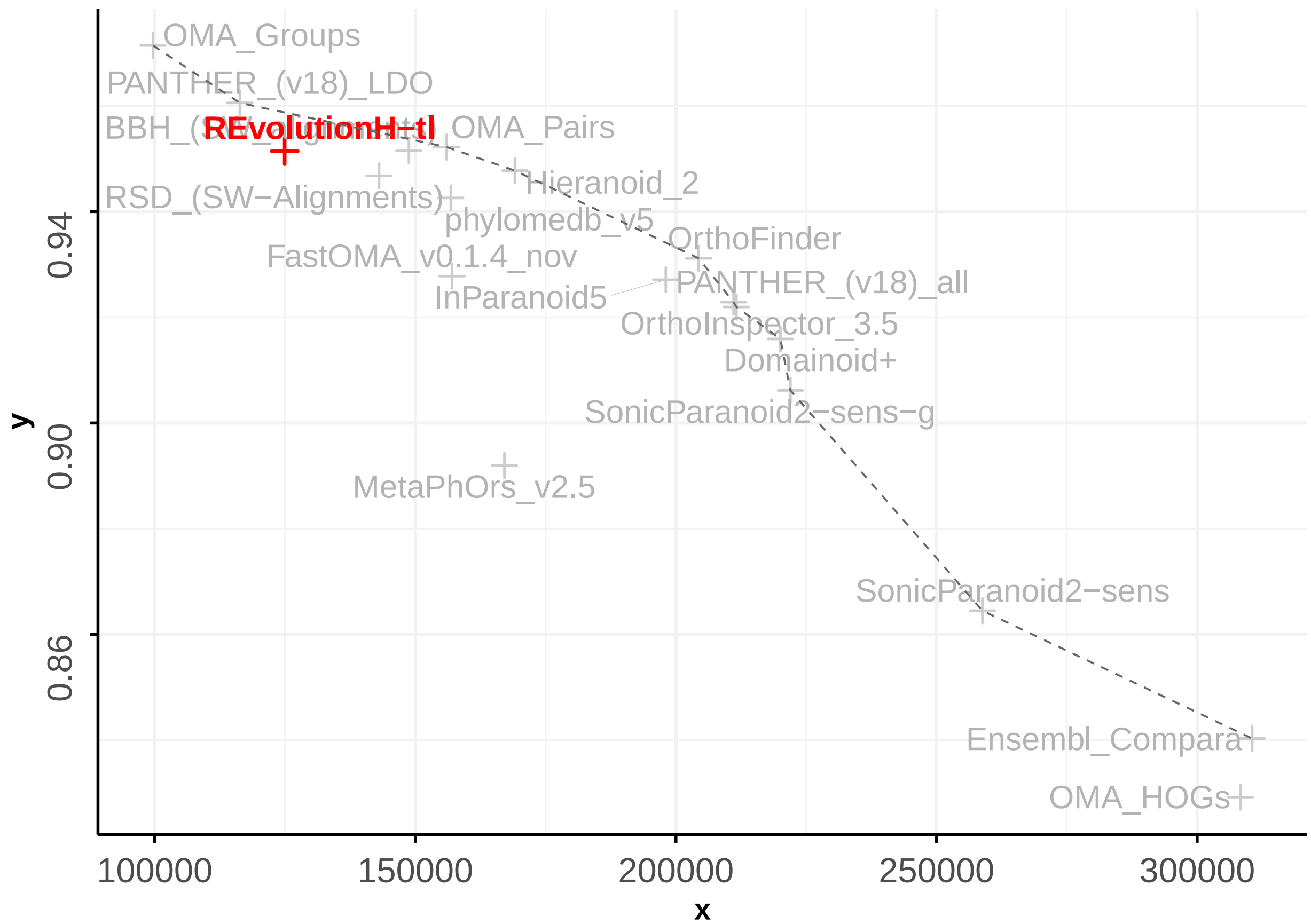

S2-Fig 13. ECtest x-axis detects recall measured as number of ortholog relations; y-axis detects precision measured as average Schlicker Similarity
