# Supplementary material for: REvolutionH-tl 2.0: A fast and robust tool for decoding evolutionary gene histories
Source: PLoS Comput Biol. 2026 Aug 12;22(8):e1013017. doi: 10.1371/journal.pcbi.1013017 (PMC13485135; doi:10.1371/journal.pcbi.1013017)
Supplement: Supplementary file 3 [file pcbi.1013017.s003.pdf]

S3 Appendix. Discordance between inferred and true phylogenies

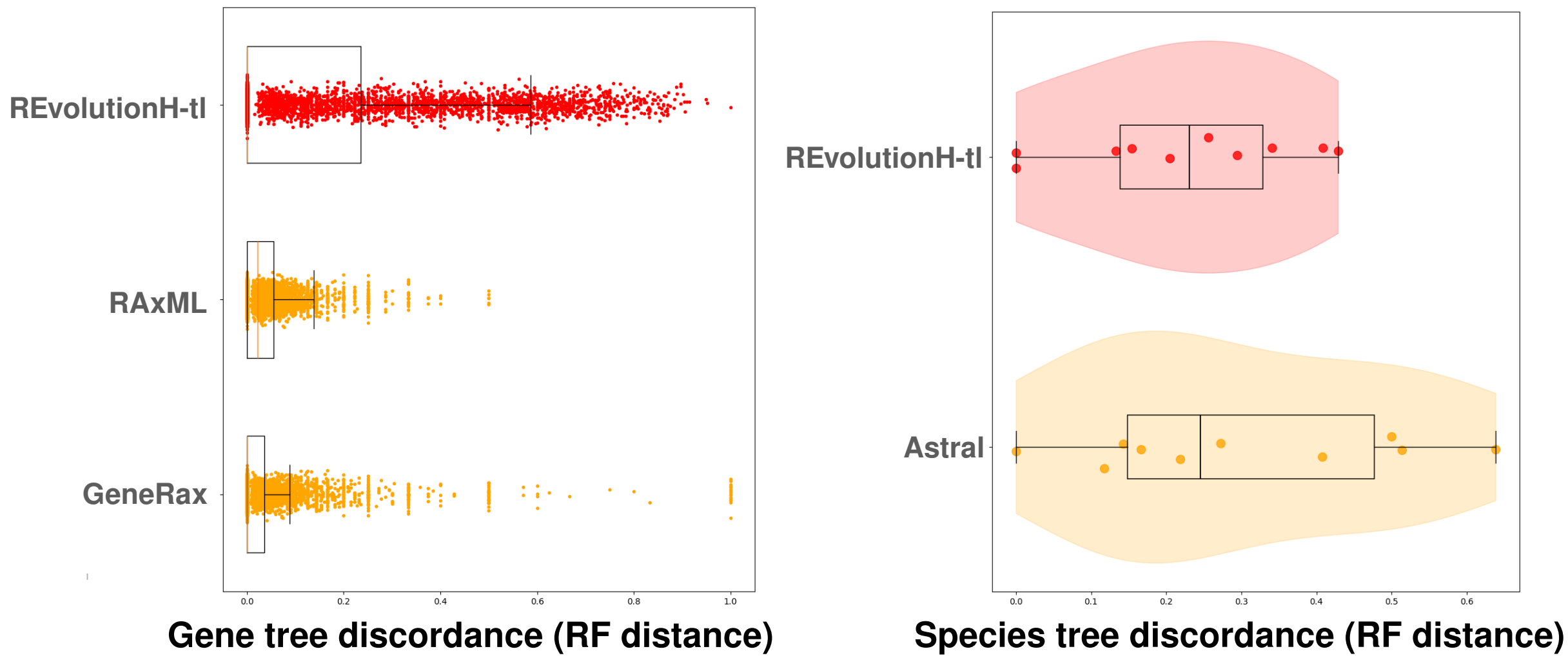

S3-Fig 1. Discordance between inferred and true phylogenies Robinson-Foulds distance is compued for the rooted trees inferred using different methodologies for gene (left) and species trees (right) on the `SaGePhy5-50` dataset. `REvolutionH-tl` gene trees correspond to the 5,736 fully recovered gene trees following reconciliation. `RaxML` shows 5,848 inferred trees, and `GeneRax` reconstructs 5,885 trees. For `RAxML` and `GeneRax`, input consists of multiple sequence alignments (MSAs) generated with `MAFFT`. Both tools also require a molecular evolution model; we used the NJ-based model configuration for consistency and rooted the trees at the midpoint of the longest path. The average for `REvolutionH-tl` is 0.144, for `RAxML` is 0.037, and for `GeneRax` is 0.144. On the right side, each dot represents a species tree with $\{5, 10, \ldots, 35, 40, 50\}$ taxa, inferred from up to 600 gene trees produced at the step 3 of `REvolutionH-tl`.
